# Stress-induced changes in social dominance are scaled by AMPA-type glutamate receptor phosphorylation in the medial prefrontal cortex

**DOI:** 10.1038/s41598-018-33410-1

**Published:** 2018-10-09

**Authors:** Min-Jung Park, Bo Am Seo, Boyoung Lee, Hee-Sup Shin, Myoung-Goo Kang

**Affiliations:** Center for Cognition and Sociality, Institute for Basic Science (IBS), Daejeon, 34141 Republic of Korea

## Abstract

The establishment and maintenance of social dominance are critical for social stability and the survival and health of individual animals. Stress lead to depression and a decrease in the social status of depressed persons is a risk factor for suicide. Therefore, we explored the mechanistic and behavioral links among stress, depression, and social dominance and found that mice subjected to chronic restraint stress (CRS), an animal model of stress-induced depression, showed decreased social dominance as measured by a dominance tube test. Importantly, this submissive behavior was occluded by the antidepressant, fluoxetine, a selective serotonin reuptake inhibitor. It is known that social dominance is controlled by synaptic efficacy in the medial prefrontal cortex (mPFC) and that AMPA-type glutamate receptor (AMPA-R) is a key molecule for synaptic efficacy. We found that the phosphorylation on AMPA-R was bidirectionally changed by CRS and fluoxetine in the mPFC of mice with CRS. Moreover, we found a strong correlation between social dominance and AMPA-R phosphorylation that regulates synaptic efficacy by modulating the synaptic targeting of AMPA-R. Our correlational analysis of the behavior and biochemistry of the CRS model suggests that AMPA-R phosphorylation in the mPFC may serve as a biomarker of social dominance related to stress.

## Introduction

Social dominance is a social structure of an animal group based on the relative social rank of its members^1,2^. Dominance status is critical for social stability and the survival and health of individual animals^3–5^. Stress and social behaviors are deeply intertwined^6^. In both humans and rodents, chronic stress induces numerous pathophysiological effects on brain function and behaviors such as depressive-like symptoms^7,8^ and a decrease in the social status of depressed patients is a risk factor for suicide^9^. It has been suggested that chronic stress could alter social dominance behavior^10^. In addition, stress is highly associated with a low rank in a social hierarchy^6,11,12^. An analytical study of the social context–dependent relationships between mouse dominance and plasma corticosterone, a stress hormone, found that subordinate males living in social hierarchies had significantly higher levels of plasma corticosterone than alpha males and pair-housed subordinate males^11^. These results suggest possible mechanistic and behavioral links among stress, depressive-like behaviors and social dominance.

The serotonergic system is involved in the modulation of social dominance and depressive-like behaviors. Elevated cortisol levels lower serotonin function in the brain leading to the depressive state^13^. Selective serotonin reuptake inhibitors (SSRIs) are the most widely prescribed antidepressants^14^. Serotonin has been shown to be closely linked to social dominance^3^. Polymorphisms of the serotonin transporter gene variation are associated with variation in the perception of social status within social hierarchies in human and non-human primates^15^. Social dominance modulates internal serotonin levels, and serotonin levels can modulate the hierarchy of vervet monkeys^3,16^. A similar effect of serotonin on social dominance of humans has also been reported. A group of people who ate meals including tryptophan (3 g/day), a precursor of serotonin, for 12 days showed a significant increase in dominant behavior compared to the control group^17^. Other neurotransmitters such as dopamine^18^, oxytocin^19^, and novel neuropeptides B and W^20^ are also known to be associated with social dominance.

Several brain regions are known to be involved in the perception and learning of social dominance, such as the amygdala, hippocampus, striatum, intraparietal sulcus, ventromedial prefrontal cortex, and lateral prefrontal cortex^21^. Specifically, the medial prefrontal cortex (mPFC) has been implicated in social cognition which is important for social hierarchy behavior^22^. Functional brain imaging in humans has shown that the dorsolateral prefrontal cortex (dlPFC) and mPFC are associated with dominance–related behaviors^15,23^. In the mouse brain, the functional homolog of the dlPFC and mPFC is the dorsomedial PFC, which includes the anterior cingulate cortex, prelimbic cortex, and infralimbic cortex^24^. Lesion of the mPFC lowers a rat’s social rank^25^. Furthermore, recent studies of dominance by Hu’s group with the dominance tube test have identified the mPFC as a control center for social dominance^26,27^. In the mPFC, excitatory synaptic efficacy is higher in dominant mice than in subordinates, and the bidirectional manipulation of synaptic efficacy in the mPFC alters social dominance^26^. The winning history alters thalamic input to the mPFC, leading to long-lasting changes in the social dominance status^27^. The functional synaptic activity in the mPFC has also been considered to be important for mediating key symptoms of depression induced by chronic social stress^28^. Therefore, the mPFC is likely a key brain region for stress, depression and social dominance.

Recent studies have implicated the AMPA-type glutamate receptor (AMPA-R) as a key molecule involved in the etiology of depression and in the action of antidepressants. In the brains of human patients with depressive symptoms (postmortem) and of rodent models of depression, the AMPA-R is significantly altered compared to control brains in terms of expression, subcellular localization, and post-translational modification. In addition to the mRNA/protein level of the AMPA-R itself, the expression of signaling molecules downstream of the AMPA-R are also affected by antidepressants^29^. The phosphorylation of the AMPA-R, which is known to be important for AMPA-R-mediated changes in the synaptic efficacy, is altered in the rodent model of depression. The phosphorylation of the AMPA-R is occluded by fluoxetine, an SSRI^29^. Conventionally, medicines that alter serotonergic and/or noradrenergic neurotransmission have been widely used as antidepressants. The SSRI fluoxetine (Prozac) has traditionally been one of the most prescribed medicines for depression and is also used as a standard antidepressant in many preclinical studies with animal models of depression. Because of the strong and reliable SSRI effects on depressive-like phenotypes such as immobility, which is simple and easy to evaluate, rodent models with stress have served in the screening of antidepressant candidates^30^. Recent, research has suggested a relationship between AMPA-R mediated neuronal activity changes in the mPFC and social dominance^26,31^.

To explore the mechanistic and behavioral links among stress, depression, and social dominance, we subjected mice to chronic restraint stress (CRS) and analyzed depressive-like phenotypes such as social behaviors. The exposure to CRS significantly affected social behaviors such as social dominance in addition to other depressive-like behaviors. We found that the social behaviors were attenuated by stress, and the attenuation was occluded by fluoxetine co-treatment. Moreover, in the mPFC, one of the main brain regions controlling social dominance, the CRS and fluoxetine treatment bidirectionally changed the phosphorylation of the AMPA-R subunits (Ser818 and Ser831 of the GluA1 subunit and Ser880 of the GluA2 subunit). The phosphorylation is known to regulate synaptic efficacy. Furthermore, we found strong correlations between social dominance and the phosphorylation of the AMPA-R (Ser818 and Ser831). Our study evaluated social dominance in a mouse model of chronic stress through a correlational analysis of behavior and biochemistry, and suggests that AMPA-R phosphorylation in the mPFC may serve as a biomarker of social dominance related to stress.

## Results

### Chronic stress induced depressive-like behaviors, which were occluded by fluoxetine co-treatment

**Figure 1:**
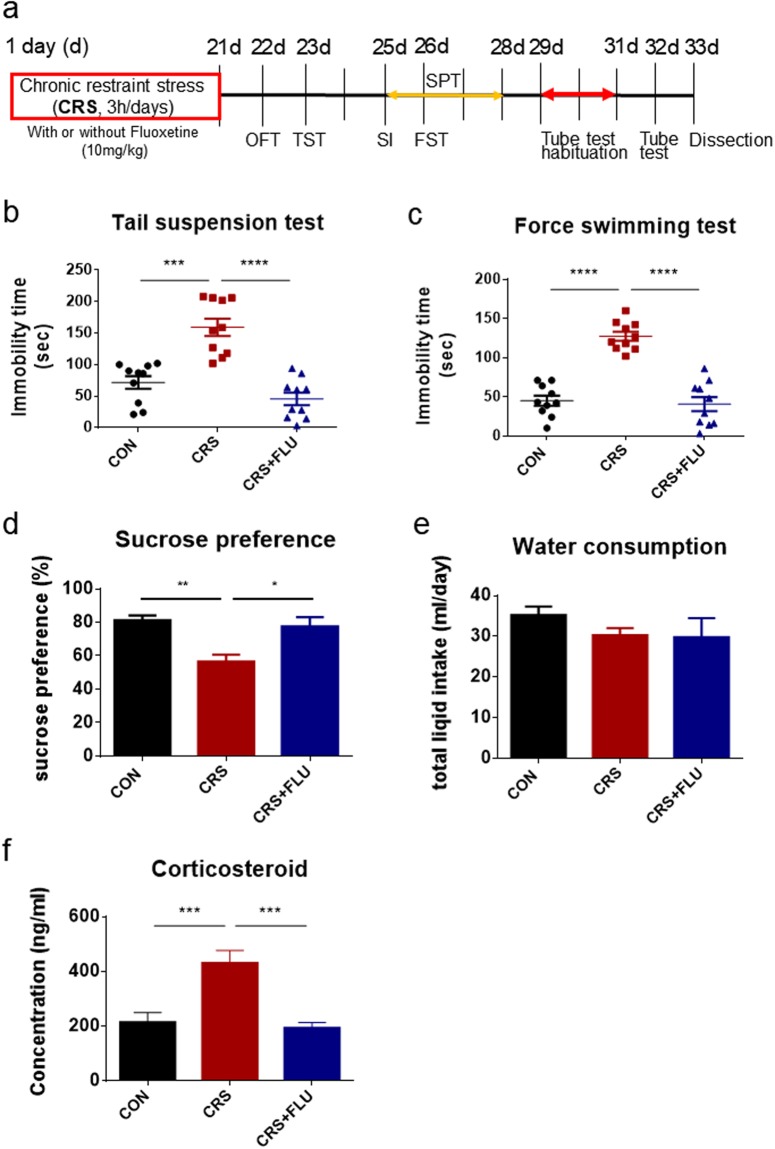
Chronic stress induced depressive-like behaviors, which were occluded by fluoxetine. (**a**) Experimental timeline of chronic restraint stress (CRS), open-field test (OFT), tail suspension test (TST), social interaction (SI), forced swimming test (FST), sucrose preference test (SPT), dominance tube test, and dissection. CRS was applied to mice for 21 days with or without fluoxetine (FLU, 10 mg/kg). Control (CON) and CRS mice were injected with saline solution. (**b**) In the TST, immobility was higher in the CRS group compared with the CON group. One-way ANOVA: F(2, 27) = 27.32, ****p < 0.0001. CON (71.80 ± 10.06, *n* = 10) vs CRS (159.3 ± 13.72, *n* = 10), ****p < 0.0001; CRS vs CRS + FLU (45.50 ± 10.02, *n* = 10), ****p < 0.0001. (**c**) In the FST, immobility was higher in the CRS group compared with the CON group. One-way ANOVA: F(2, 27) = 46.59, ****p < 0.0001. CON (45.00 ± 6.393, *n* = 10) vs CRS (127.4 ± 5.747, *n* = 10), ****p < 0.0001; CRS vs CRS + FLU (40.60 ± 8.947, *n* = 10), ****p < 0.0001. (**d**) In the SPT, sucrose preference was significantly reduced in the CRS group compared with the CON group. One-way ANOVA: F(2, 9) = 9.433, *p = 0.0062. CON (81.31 ± 2.841, *n* = 10) vs CRS (56.56 ± 4.068, *n* = 10), **p = 0.0059; CRS vs CRS + FLU (77.46 ± 5.642, *n* = 10), *p = 0.0155. Consumption of water or 1% sucrose solution was measured for 2 days after 48 h of habituation of the two bottle conditions. (**e**) Total water consumption was not different between all groups during the SPT. One-way ANOVA: F(2, 9) = 0.9244, *p* = 0.4314. CON (35.25 ± 4.193, *n* = 10); CRS (39.25 ± 1.797, *n* = 10); CRS + FLU (29.75 ± 4.732, *n* = 10). (**f**) Corticosterone level in serum was analyzed after termination of the behavior tests by enzyme-linked immunosorbent assay. One-way ANOVA: F(2, 27) = 13.55, ****p < 0.0001. CON (213.7 ± 36.14, *n* = 10) vs CRS (431.3 ± 46.10, *n* = 10), ***p = 0.0004; CRS vs CRS + FLU (193.4 ± 20.47, *n* = 10), ***p = 0.0001. (**b**–**d**,**f**) FLU treatment occluded the CRS effect on the TST, FST, and corticosterone level. Post hoc Bonferroni’s multiple comparisons revealed a significant difference between the three groups. Data are shown as mean ± SEM.

To induce depressive-like behavior, we treated mice with CRS, followed by a series of behavioral analyses of depressive-like phenotypes and dissection of mice for measurement of stress hormones in serum and harvesting of brain tissues (Fig. 1a). To induce chronic stress, two groups of mice were subjected to CRS (3 h per day for 21 days). In one group, fluoxetine was administered daily by intraperitoneal injection (10 mg/kg per day) 30 min before the restraint session for up to 3 weeks (CRS + FLU). As a negative control group, a group of mice was kept in cages with restriction of food and water for 3 h per day for 21 days after intraperitoneal injection of saline solution (CON). The sham injection was also applied to the CRS group. One day after the final CRS, all groups of mice were given an open-field test (OFT), which showed no difference in locomotion activity (Supplementary Fig. S1a,b ; one-way analysis of variance (ANOVA) with Bonferroni’s post hoc test, F (2, 27) = 0.1111, *p* = 0.8953) and anxiety (Supplementary Fig. S1c; F (2, 27) = 1.880, *p* = 0.1720) among the three groups. We examined the induction of depression by chronic stress by analyzing depressive-like behaviors with the tail suspension test (TST, Fig. 1b), the forced swimming test (FST, Fig. 1c), and the sucrose preference test (SPT, Fig. 1d). The results show that the CRS significantly increased immobility (Fig. 1b; F (2, 27) = 27.32, ****p < 0.0001, Fig. 1c; F (2, 27) = 46.59, ****p < 0.0001) and anhedonia (Fig. 1d; F (2, 9) = 9.433, **p = 0.0062), which are two distinct depressive phenotypes in rodents. There was no difference in water consumption (Fig. 1e; F (2, 9) = 0.9244, *p* = 0.4314), indicating that the reduced sucrose preference associated with CRS was not due to a decrease in thirst or other physiological reasons. The higher level of corticosterone in the CRS group than in the control group (Fig. 1f; F (2, 27) = 13.55, ****p < 0.0001) confirmed the induction of stress by CRS and the prolonged effect of chronic stress during the period of behavioral analyses (days 22 to 33). It is known that the CRS effect was prolonged for a minimum of 4 weeks^32^. Moreover, the CRS effects were all occluded by fluoxetine co-treatment, suggesting that the CRS effect on these behaviors could be controlled by the serotonergic system in the brain.

### Chronic stress decreased sociability and social novelty preference, which was occluded by fluoxetine co-treatment

**Figure 2:**
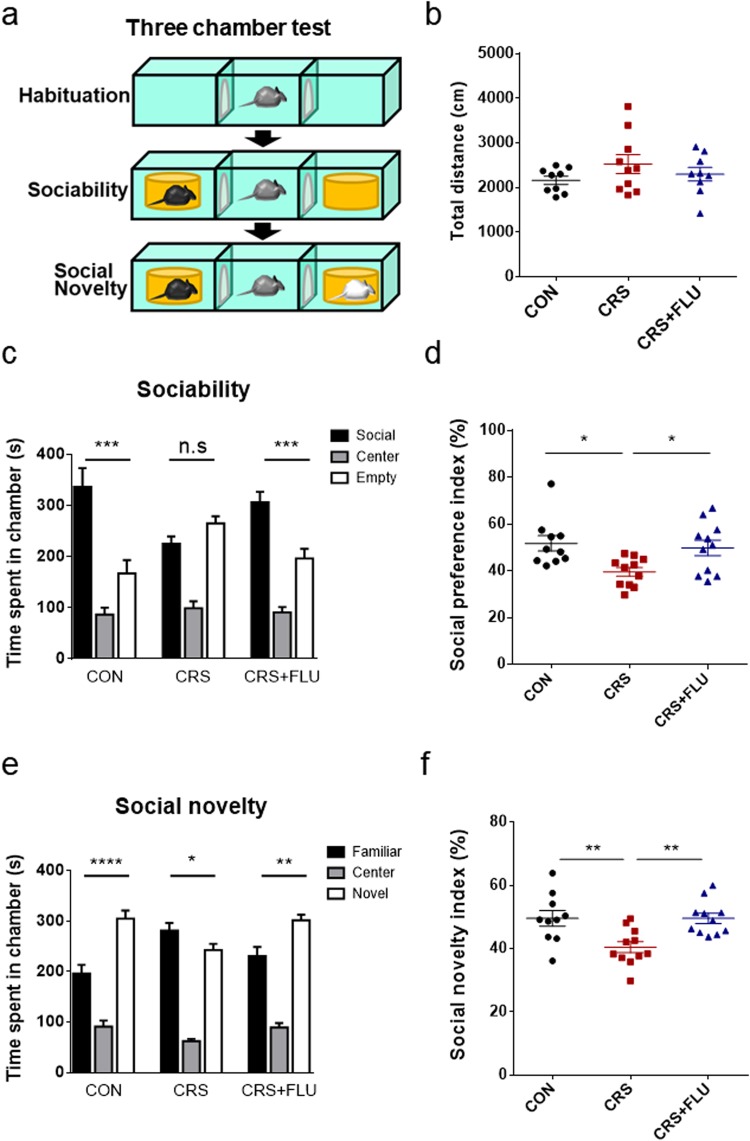
Chronic stress impaired sociability and social novelty preference, which were occluded by fluoxetine. (**a**) Schema of the three-chamber test explaining procedure of three 10-min sessions. (**b**) Locomotion in three chambers was measured during habituation session (session 1), which showed no difference between groups. One-way ANOVA: F(2, 25) = 1.302, *p* = 0.2897. CON (2162 ± 91.46, *n* = 9); CRS (2526 ± 209.2, *n* = 10); CRS + FLU (2298 ± 151.9, *n* = 9). (**c**) During sociability session (session 2), CON and CRS + FLU groups spent more time in social chamber than in empty chamber. However, the CRS group spent almost the same amount of time in the two chambers. One-way ANOVA: CON group; F(2, 24) = 21.97, ****p < 0.0001), social chamber (336.7 ± 36.94, *n* = 9) *vs* empty chamber (167.2 ± 26.03, *n* = 9), ***p = 0.0004. CRS group; F(2, 27) = 38.03, ****p < 0.0001, social chamber (225.1 ± 14.85, *n* = 10) vs empty chamber (265.4 ± 13.76, *n* = 10), *p* = 0.0956. CRS + FLU group; F(2, 24) = 39.94, ****p < 0.0001, social chamber (307.0 ± 20.10, *n* = 9) vs empty chamber (196.7 ± 18.93, *n* = 9), ***p = 0.0003. (**d**) Social preference index was calculated by dividing the time spent in social chamber by total time spent, and expressing it as a percentage. CRS decreased this index significantly, and FLU occluded it. One-way ANOVA: F(2, 24) = 6.261, *p = 0.0065. CON (51.03 ± 3.930, *n* = 8) vs CRS (37.52 ± 2.475, *n* = 10), *p = 0.0137; CRS vs CRS + FLU (51.16 ± 3.350, *n* = 9), *p = 0.0102. (**e**) During social novelty session (session 3), CON and CRS + FLU groups spent more time in novel chamber than in familiar chamber. However, the CRS group spent almost the same amount of time in the two chambers. One-way ANOVA: CON group; F(2, 24) = 49.73, ****p < 0.0001, familiar chamber (196.4 ± 16.97, *n* = 9) *vs* novel chamber (305.1 ± 16.02, *n* = 9), ****p < 0.0001. CRS group; F(2, 27) = 101.5, ****p < 0.0001, familiar chamber (281.4 ± 15.19, *n* = 10) vs novel chamber (242.8 ± 12.44, *n* = 10), *p = 0.0480. CRS + FLU group; F(2, 24) = 65.95, ****p < 0.0001, familiar chamber (230.9 ± 18.15, *n* = 9) vs novel chamber (301.8 ± 11.16, *n* = 9). (**f**) Social novelty index was calculated by dividing the time spent in novel chamber by total time spent, and expressing it as a percentage. CRS decreased this index significantly, and the index change was occluded by FLU. One-way ANOVA: F(2, 25) = 7.154, **p = 0.0035. CON (50.85 ± 2.670, *n* = 9) vs CRS (40.47 ± 2.073, *n* = 10), **p = 0.0053; CRS vs CRS + FLU (50.30 ± 1.860, *n* = 9), **p = 0.0083. (**c**–**f**) Post hoc Bonferroni’s multiple comparisons revealed a significant difference between three groups (or chambers). Data are shown as mean ± SEM.

A symptom of depression induced by stress is impairment in social functioning. It is also known that the negative symptoms of sociability, such as social anhedonia and avoidance of social interaction (SI), are observed in mouse models of depression induced by stress^33^. We tested whether CRS induced changes in social behavior, sociability, and social novelty (Fig. 2).

The sociability test was performed through three consecutive sessions for 10 min each using three-chambered boxes (Fig. 2a). Session 1 was the habituation period, during which the mice were allowed to freely move around chambers for 10 min. During habituation, there was no difference in the total distance of movement among the groups (Fig. 2b; F (2, 25) = 1.302, *p* = 0.2897). To measure the preference for sociability, mice were next placed in the middle chamber after an unfamiliar mouse in a cup had been placed in one of the two side chambers (social chamber; Fig. 2a). For 10 min of session 2, the control mice spent more time in the social chamber than in the empty chamber (Fig. 2c; CON, F (2, 24) = 21.97, ****p < 0.0001). However, the amounts of time the CRS mice spent in the two side chambers did not differ significantly (Fig. 2c; CRS, F (2, 27) = 38.03, ****p < 0.0001). Fluoxetine co-treatment occluded the impairments in sociability in the CRS group (Fig. 2c; CRS + FLU, F (2, 24) = 39.94, ****p < 0.0001). The social preference index (the percentage of time spent in the social chamber during the 10 min) was significantly lower in the CRS group than in the control groups (Fig. 2d; F (2, 24) = 6.261, **p = 0.0065). In session 3, we tested social novelty. An unfamiliar mouse in a cup was placed in one of the two side chambers (novel chamber), and a familiar mouse from session 2 was placed in another side chamber (familiar chamber; Fig. 2a). During the 10 min of session 3, the control group spent significantly more time in the novel chamber than in the familiar chamber (Fig. 2e; CON, F (2, 24) = 49.73, ****p < 0.0001). However, the amounts of time the CRS mice spent in the two side chambers did not differ significantly (Fig. 2e; CRS, F (2, 27) = 101.5, ****p < 0.0001). Fluoxetine co-treatment again occluded the impairments in social novelty (Fig. 2e; CRS + FLU, F (2, 24) = 65.95, ****p < 0.0001). The social novelty index, the percentage of time spent in the novel chamber during the 10 min, was significantly lower in the CRS group than in the control groups (Fig. 2f; F (2, 25) = 7.154, **p = 0.0035). These results demonstrate that chronic stress impaired sociability and social novelty preference and suggest that the stress-induced changes in the social behavior is modulated by the serotonergic system.

### Social dominance was bidirectionally altered by stress and fluoxetine, and was highly correlated with depressive-like phenotypes

To determine whether chronic stress affected social dominance, we decided to use the dominance tube test. This test has become popular as a standard test to measure the dominance that underlies the social hierarchy of mice and rats for several reasons. The dominance tube test is a simple and reliable behavioral test that does not include a violent conflict situation. Basically, the only essential equipment is a narrow cylindrical tube through which a rodent can pass. Consistent results can be expected from day-to-day, and the dominance derived from the tube test is reproducible in other hierarchy tests. Furthermore, the dominance tube test can be used not only for familiar cage-mates from the same strain but also for unfamiliar non-cage-mates^26,34^.

**Figure 3:**
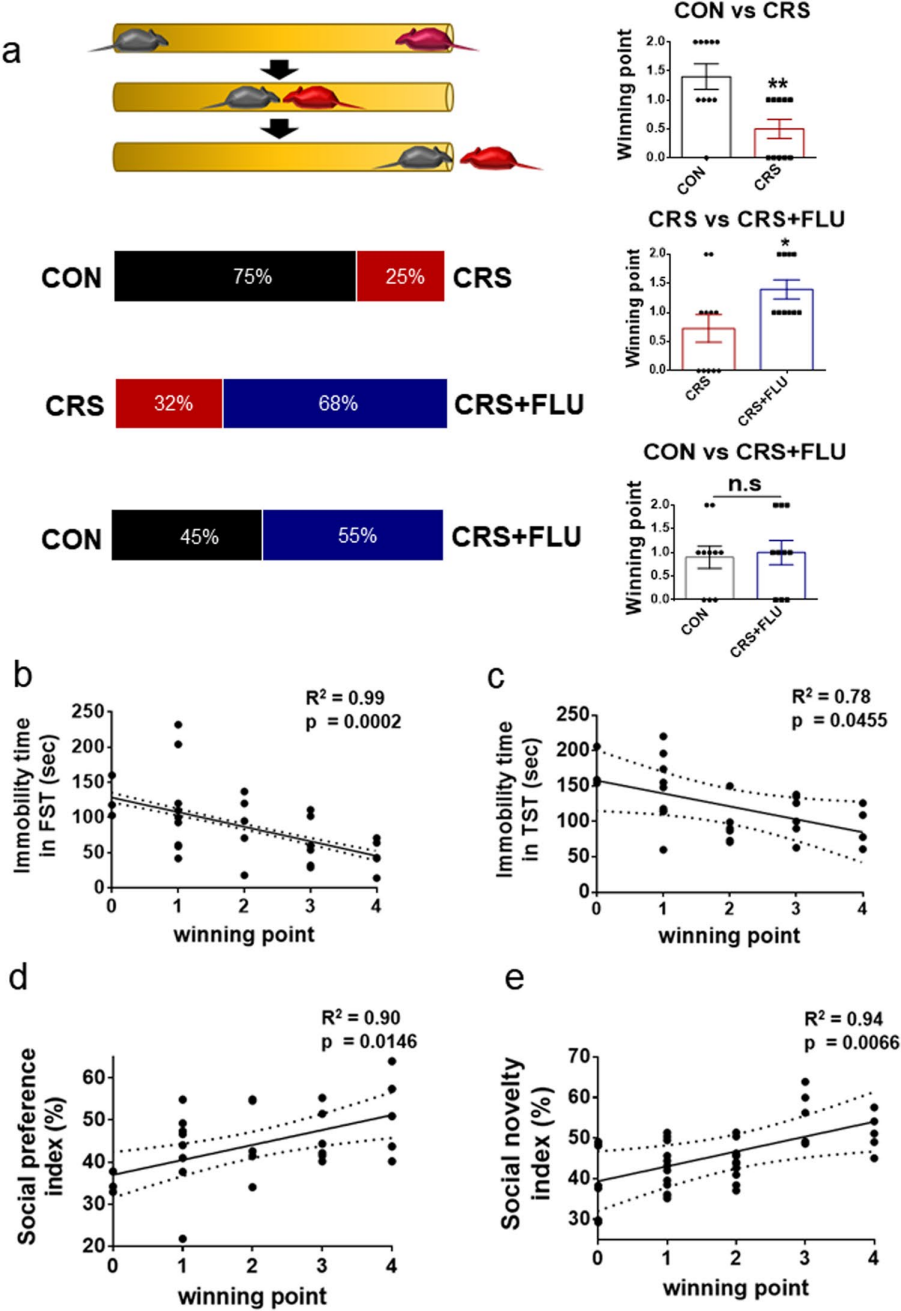
Social dominance was bidirectionally altered by stress and fluoxetine, and highly correlated with depressive phenotypes. (**a**) Schema of dominance tube test showing competition of two mice in a 30-cm cylindrical tube. The horizontal bar graph shows percentages of wins in the tube test. CON group (75%) showed higher win scores against the CRS group (25%). The CRS group (32%) showed lower win scores against CRS + FLU group (68%). The CON group (45%) showed about half wins against the CRS + FLU group (55%). Winning points were calculated by counting win number for each mouse, averaged and presented histogram. The winning points of the CRS group were significantly lower compared with those of the CON and CRS + FLU. CON (1.400 ± 0.2211, *n* = 10) vs CRS (0.500 ± 0.1667, *n* = 10), **p = 0.0044, Student’s *t*-test. CRS (0.7273 ± 0.2371, *n* = 10) vs CRS + FLU (1.400 ± 0.1633, *n* = 10), *p = 0.0336, Student’s *t*-test. CON (0.900 ± 0.2333, *n* = 10) vs CRS + FLU (1.000 ± 0.2582, *n* = 10), *p* = 0.7771, Student’s *t*-test. (**b**–**e**) High correlation of winning points with the FST (a negative linear regression, R^2^ = 0.99, *n* = 30, ***p = 0.0002. Winning points were calculated by counting win number in the total of four matches for each mouse and averaged. (**b**) TST (a negative linear regression, R^2^ = 0.78, *n* = 30, *p = 0.0455) (**c**), sociability (a positive linear regression, R^2^ = 0.90, *n* = 27, *p = 0.0146) (**d**) and social novelty (a positive linear regression, R^2^ = 0.94, *n* = 28, *p = 0.0066) (**e**) indicate strong correlation between social dominance and those depressive phenotypes. Data are shown as mean ± SEM.

Before the test trials, all mice were trained to adapt to the testing tube by being guided to traverse a testing tube in alternating directions for three successive days. We confirmed that all mice were able to pass through the tube freely. After training and habituation, unfamiliar mice from different cages were placed at opposite ends of the test tube and released so that the mice were able to meet in the middle of the tube. During the match, if a mouse pushed another mouse through the test tube, it was scored as a win. Each mouse was subjected to a total of four matches, and a mouse’s dominance was calculated as the percentage of wins out of the four matches (Fig. 3a). The CRS group had a lower percentage of wins against the CON group and the CRS + FLU group. However, the percentage of wins was almost the same between the CON group and the CRS + FLU group (Fig. 3a). For statistical analysis, the winning points for each group were calculated by counting the number of wins in a match (e.g., 0, 1, 2) to compare the group differences. The CRS group had significantly fewer winning points than the control group (Fig. 3a; Student’s *t*-test, **p = 0.0044), suggesting that stress decreased social dominance. Fluoxetine occluded the CRS effect on social dominance (Fig. 3a**;** Student’s *t*-test, *p = 0.0336). Because the three groups of mice differed in body weight (Supplementary Fig. S1d ; F (2, 27) = 5.081, *p = 0.0134), we tried to match two mice with a similar body weight for the dominance tube test to reduce the potential effects of body weight on winning. For the weight-matching condition, some mice could not be used for the tube test. The dominance and submissive behaviors of each group from the tube test were confirmed by another dominance test, the urine marking test (Supplementary Fig. S2)

We also analyzed the correlation of the winning points for total matches (e.g., 0, 1, 2, 3, 4) from all groups with the results of the FST (R^2^ = 0.99, ***p = 0.0002), TST (R^2^ = 0.78, *p = 0.0455), social preference (R^2^ = 0.90, *p = 0.0146), and social novelty (R^2^ = 0.94, *p = 0.0066; Fig. 3b–e). We found strong correlations among winning points and depressive-like behaviors induced by stress, supporting our hypothesis that the decrease in social dominance was induced by chronic stress as were the other depressive-like phenotypes.

Using a well-standardized mouse model of chronic stress, we demonstrated that chronic stress induced alterations in social dominance and that the stress-induced changes of social dominance were occluded by fluoxetine, which regulates the serotonergic system in the brain.

### Phosphorylation of AMPA-R was bidirectionally altered by stress and fluoxetine, and was highly correlated with social dominance

To identify the molecular alterations that underlie the bidirectional changes in social dominance associated with chronic stress and fluoxetine, we decided to analyze the AMPA-R phosphorylation in the mPFC. The mPFC has been known as a region involved in social dominance^15,23,25^. Hu’s group clearly demonstrated that the mPFC is one of the chief brain regions controlling social dominance by manipulating the synaptic strength of the mPFC through a bidirectional alteration of synaptic expression of the AMPA-R^26,27^. Synaptic targeting of the AMPA-R is regulated by phosphorylation on AMPA-R subunits, resulting in the regulation of synaptic efficacy^35^. We hypothesized that the submissive behavior of the CRS mice was related to AMPA-R phosphorylation in the mPFC, and we analyzed the major phosphorylation sites on AMPA-R subunits GluA1 and GluA2, which are known to be involved in changes of synaptic efficacy: Ser818, Ser831, and Ser845 on GluA1 and Ser 880 on GluA2.

**Figure 4:**
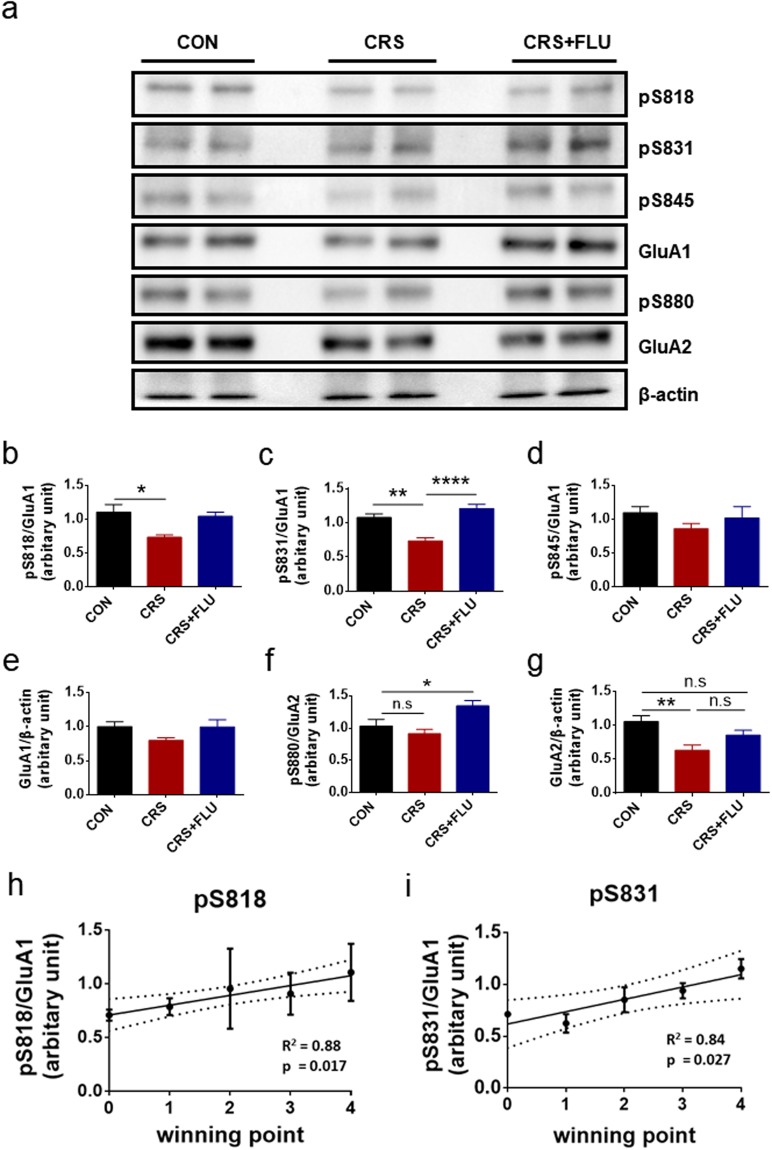
Phosphorylation of AMPA-R was bidirectionally altered by stress and fluoxetine, and highly correlated with social dominance. (**a**) Representative data from western blot analyses of AMPA-R subunits and their phosphorylation in the mouse mPFC. (**b**–**g**) Quantification of the western blot analyses of AMPA-R subunits and their phosphorylation demonstrated: significant decrease of the serine phosphorylation (pS) on 818 and 831 of GluA1, significant increase of the pS on 880 of GluA2 by fluoxetine, and significant decrease of the GluA2 by CRS. (**b**) Quantification of the western blot analyses of pS818. One-way ANOVA: F(2, 12) = 4.390, *p = 0.0371. CON (1.106 ± 0.1125, *n* = 5) vs CRS (0.7343 ± 0.0367, *n* = 5), *p = 0.0271; CRS vs CRS + FLU (1.046 ± 0.0602, *n* = 5), *p* = 0.1470. (**c**) Quantification of the western blot analyses of pS831. One-way ANOVA: F(2, 12) = 20.38, ***p = 0.0001. CON (1.078 ± 0.05397, *n* = 5) vs CRS (0.7341 ± 0.04652, *n* = 5), **p = 0.0015; CRS vs CRS + FLU (1.209 ± 0.06152, *n* = 5), ****p < 0.0001. (**d**) Quantification of the western blot analyses of pS845. One-way ANOVA: F(2, 12) = 0.09224, *p* = 0.9125. CON (1.096 ± 0.09151, *n* = 5) vs CRS (0.8616 ± 0.07809, *n* = 5), *p* = 0.9118; CRS vs CRS + FLU (1.029 ± 0.1740, *n* = 5), *p* = 0.5955. (**e**) Quantification of the western blot analyses of GluA1. One-way ANOVA: F(2, 12) = 2.226, *p* = 0.1506. CON (1.000 ± 0.06877, *n* = 5) vs CRS (0.7979 ± 0.0400, *n* = 5), *p* = 0.1688; CRS vs CRS + FLU (0.9941 ± 0.1074, *n* = 5), *p* = 0.1852. (**f**) Quantification of the western blot analyses of pS880. One-way ANOVA: F(2, 12) = 6.772, *p = 0.0107. CON (1.032 ± 0.1031, *n* = 5) vs CRS (0.9127 ± 0.06751, *n* = 5), *p* = 0.5320; CON vs CRS + FLU (1.344 ± 0.08238, *n* = 5), *p = 0.0435. (**g**) Quantification of the western blot analyses of GluA2. One-way ANOVA: F(2, 12) = 7.165, **p = 0.0090. CON (1.056 ± 0.08569, *n* = 5) vs CRS (0.6314 ± 0.07982, *n* = 5), **p = 0.0052; CRS vs CRS + FLU (0.8552 ± 0.07211, *n* = 5), *p* = 0.1874; CON vs CRS + FLU, *p* = 0.2675. (**h**,**i**) The phosphorylation on S818 and S831 is highly correlated with winning points in the tube test. pS818 vs winning points (a positive linear regression, R^2^ = 0.88, *n* = 14, *p = 0.017) and pS831 vs winning points (a positive linear regression, R^2^ = 0.84, *n* = 14, *p = 0.027). (**b**,**c**,**f**,**g**) Post hoc Bonferroni’s multiple comparisons revealed a significant difference between three groups. Data are shown as mean ± SEM.

After the behavioral analyses described above, the mPFC was dissected out from each mouse brain and used for biochemical analysis of AMPA-R phosphorylation. As shown in the representative western blots (Fig. 4a) and quantification (Fig. 4b–g), the CRS significantly decreased the phosphorylation on Ser818 (pS818) and Ser831 (pS831) of GluA1 (Fig. 4b; F (2, 12) = 4.390, *p = 0.0371, Fig. 4c; F (2, 12) = 20.38, ***p = 0.0001). The effects of CRS on pS818 and pS831 were occluded by fluoxetine, although the reversal was statistically significant only for pS831 (Fig. 4b,c). The phosphorylation on S845 (pS845) and total GluA1 were not significantly changed by CRS or fluoxetine, but there were small changes similar to that of pS818 and pS831 (Fig. 4d; F (2, 12) = 0.09224, *p* = 0.9125). The phosphorylation on S880 (pS880) of GluA2 was significantly changed only by fluoxetine (Fig. 4f; F (2, 12) = 6.772, *p = 0.0107). In contrast, the total GluA2 was significantly decreased by the CRS and was not affected by fluoxetine (Fig. 4g; F (2, 12) = 7.165, **p = 0.0090). Because the increase of pS818 and pS831 is essential for the synaptic targeting of GluA1^36–38^, the decrease of pS818, pS831, and GluA2 by CRS would decrease the number of synaptic AMPA-R, resulting in low synaptic efficacy in the mPFC. However, the increase of pS818 and pS831 by fluoxetine would increase the number of synaptic AMPA-R, resulting in an occlusion of the CRS effect on the synaptic efficacy in the mPFC. Our correlation analysis showed that the two phosphorylation sites on S818 (R^2^ = 0.88, *p = 0.017) and S831 (R^2^ = 0.84, *p = 0.027) of the GluA1 subunit of AMPA-R were highly correlated with winning points from the social dominance test (Fig. 4h,i). However, the correlations of winning points with the GluA1, GluA2, pS845, and pS880 sites were not statistically significant (Supplementary Fig. S3). These biochemical analyses of the CRS mouse mPFC and correlational studies with social dominance suggest that the phosphorylation of AMPA-R (pS818 and pS831) may contribute as a biomarker for social dominance changes induced by chronic stress and fluoxetine.

## Discussion

To understand the molecular and cellular mechanisms of stress-induced changes in social dominance, we sought stress-specific molecular alterations in brain regions that could be occluded with a drug. Based on results from previous studies, we hypothesized that the AMPA-R in the mPFC is a key molecule for controlling changes in social dominance induced by chronic stress and administration of an SSRI. We tested our hypothesis using a well-established mouse model of stress-induced depression, CRS. Indeed, our data demonstrate that social dominance was bidirectionally changed by stress and the SSRI fluoxetine (Fig. 3a), which accompanied changes in three phosphorylation sites on AMPA-R subunits in the mPFC (Fig. 4a–c,f). The AMPA-R phosphorylation was highly correlated with social dominance (Fig. 4h,i) and social behaviors that were induced by chronic stress (Supplementary Fig. S4). Altogether, our results suggest that the AMPA-R phosphorylation in the mPFC may serve as a biomarker for the bidirectional changes of social dominance brought by stress and fluoxetine. Therefore, our study could contribute to the design of a simple and efficient platform that could be used for the development of new drugs for the management of social dominance in depressed patients. For example, libraries of chemicals or molecules could be screened by analyzing the changes of AMPA-R phosphorylation in mPFC neurons cultured in 96 wells, followed by a second round of behavioral screening using dominance tube tests of stressed mice injected with the candidate drugs.

We found bidirectional changes of phosphorylation of the AMPA-R subunits GluA1 and GluA2 as molecular mechanisms underlying social dominance (Fig. 4). In our study, pS818 and pS831 of GluA1 were significantly decreased by CRS. The increases in pS818 and pS831 are necessary for the synaptic incorporation of AMPA-R during long-term potentiation (LTP)^36–38^. LTP is the main mechanism for the increase of synaptic efficacy in many regions of the brain such as the mPFC and hippocampus^39^. Thus, it is likely that the decrease of pS818 and pS831 decreased the synaptic efficacy in the mPFC, resulting in a decrease of social dominance (Fig. 3a). In contrast, fluoxetine returned the pS818 and pS831 back to normal levels in the CON group (Fig. 4c), which is likely a mechanism for the recovery of social dominance in the CRS + FLU group (Fig. 3a). In agreement with our findings, the SSRI tianeptine significantly increased pS831 in the frontal cortex and in the CA3 region of the hippocampus^40^. Fluoxetine also significantly increased the pS880 of GluA2 (Fig. 4f), which could be a mechanism for the recovery of social dominance in the CRS + FLU group (Fig. 3a). Recent studies indicated that antidepressant drugs modulate synaptic plasticity that enables behavioral changes, in addition to well-known effects on depressive states or depressive-like behaviors^41^. The increase of pS880 is involved in the internalization of GluA2 from the cell surface^42,43^, and the internalization of GluA2 could increase the calcium-permeable AMPA-R (CP-AMPA-R) because GluA2 blocks calcium influx through the AMPA-R. The increase of calcium influx at the synapse by the CP-AMPA-R could positively influence the signaling pathway involved in the synaptic plasticity^44^. For example, an increase of synaptic calcium influx activates kinases such as the CaMKII, resulting in enhanced synaptic efficacy^45^. Indeed, the CP-AMPA-R is involved in the induction of LTP^46^. Therefore, it is possible that chronic administration of fluoxetine increased synaptic plasticity through the increase of pS880 in AMPAR, which allowed reversal of the CRS effect on social dominance. Interestingly, only pS831 was significantly changed by CRS and fluoxetine. The other phosphorylation sites (pS818 and pS880) were significantly changed in only one direction by either CRS or fluoxetine (Fig. 4b,c,f). This difference in direction means that the physiological status of the brain in dominant and submissive animals is determined by more than one factor or signaling pathway. This result could mean that a submissive status could be changed into a dominant status even though the cause of the submissive status (stress) remains. To explore this possibility further, we performed additional experiments that can test if fluoxetine has its own effect on animal behavior and AMPA-R phosphorylation (Supplementary Figs S5 and S6). Fluoxetine was able to alter dominance, as well as, depressive-like behaviors (TST and FST). It has been shown that depressive-behavior in rodents can be relieved by fluoxetine treatment^30,47^. The significant increase of dominance induced by fluoxetine (Supplementary Fig. S5) demonstrated that fluoxetine has its own effect on dominance. Moreover, phosphorylation of AMPA-R was also altered by fluoxetine (Supplementary Fig. S6), which indicated that fluoxetine has its own effect on AMPA-R phosphorylation at mPFC. These results strongly suggest that the modulation of AMPA-R phosphorylation in mPFC is a critical factor to change social dominance either by stress or fluoxetine. Furthermore, we tested if intra-mPFC injection of an AMPA receptor blocker (NBQX) could antagonize the effects of fluoxetine. Dominance was significantly reduced by NBQX injection regardless of fluoxetine treatment (Supplementary Fig. S7). Altogether, these additional experiments (Supplementary Figs S5–S7) strongly support our hypothesis that AMPA-R phosphorylation in the mPFC serve as a biomarker of social dominance related to stress.

Previous studies of AMPA-R phosphorylation related to stress-induced depression and SSRI administration have focused mostly on the hippocampus and found changes only on pS845. AMPA-R phosphorylation in the hippocampus has been analyzed previously with a combination of stress and fluoxetine and shows no significant changes except pS845^48^. With fluoxetine, the phosphorylation of S845 in the hippocampus decreased under the stress condition in one study^48^ but increased in another study^49^. Similarly, imipramine (the classic tricyclic antidepressant) and the SSRI tianeptine increased pS845 in the hippocampus^40,50^.

Recently, Yang *et al*. reported that chronic unpredictable mild stress (CUMS) increases aggression and enhances social dominance^51^. In their study, fluoxetine prevents aggression without reducing social dominance. Aggression and dominance are not identical concepts, as mentioned in their discussion section. In many cases, aggression and dominance are not well correlated. Moreover, there are cases in which aggression and dominance change in opposite directions^34^. Indeed, the correlation between aggressiveness and social rank in the dominance tube test has not always been apparent. For example, the highest scoring mice from the dominance tube test did not appear to be more aggressive^52^. The opposite effects of chronic stress on social dominance between our study and that of Yang’s group are not easy to explain. The differences could be due to the different methods used to induce chronic stress; we used CRS, and Yang and colleagues used CUMS. The different stress-induction methods could result in differences in social dominance. Specifically, the difference in the fluoxetine effect on social dominance could be due to the timing of treatment; we pre-treated our mice, whereas Yang and colleagues administered the drug as a post-treatment. Yang’s study concluded that social dominance is regulated by the dopaminergic system in the hippocampus based on the effect of clozapine on hippocampal microtubule-associated protein 2 and on aggression and social dominance. Clozapine is a nonspecific tricyclic antipsychotic drug that is known to affect several neurotransmitter receptors such as the GABA_B_ receptor, NMDA receptor, and serotonin and dopamine receptors^53–55^. Due to the non-specificity of clozapine, its mechanism of action is not clear. In contrast, our findings on the effects of fluoxetine on social dominance are consistent with a previous study. Fluoxetine has been found to increase the social dominance of submissive rats in the paradigm of dominant-submissive relationships^56^.

Stress is an important modulator of social behaviors. Stress generally helps organisms to deal with situations that challenge their survival, and overcome threats to homeostasis^57^. However, sustained stress can have numerous pathophysiological effects on brain function and behavior^57^. In this study, we found that chronic stress lowered social dominance as well as triggering depressive-like behaviors in rodents. We also observed impaired sociability (Fig. 2c,d) and social novelty (Fig. 2e,f) in SI tests. Social preference and social novelty are positively correlated with social dominance (Fig. 3d,e), suggesting that CRS in this study did induce adverse effects on social behaviors. Interestingly, a level of corticosterone in serum, which was elevated in the CRS group displaying submissiveness, was occluded by fluoxetine co-treatment (Fig. 1f). Consistent with our finding, it has been known that acute increases in glucocorticoids promote aggressiveness in socially challenged animals, but chronic surges in glucocorticoids produce the opposite effect^58^. Plasma glucocorticoids decrease in the winning animals, but increase in the losers^59,60^. In addition, administration of glucocorticoids to a variety of species demonstrated that elevated levels of glucocorticoids decrease social dominance and promote submissiveness^58,61–63^. It is unclear how chronic stress induced corticosterone-triggered changes in social dominance. Generally, stress activates the hypothalamo-pituitary-adrenal axis resulting in a release of corticosterone (in most rodents) or cortisol (humans) from the adrenal glands. The released hormones then enter the brain and bind to two subtypes of their receptors, the mineralocorticoid receptor and glucocorticoid receptor^64^. One recent study has shown that glucocorticoid signaling in the mPFC plays an important role in stress-mediated changes in social dominance by inhibiting the action of glucocorticoid receptors^31^. Whether glucocorticoid signaling is directly related to the changes in social dominance and impairment in SI induced by our chronic stress model and whether it is associated with the phosphorylation status of AMPA-R in mPFC remain to be examined.

Our study demonstrated stress-mediated changes in social dominance using a mouse model of depression. We identified bidirectional changes in specific phosphorylation sites on AMPA-R in the mPFC induced by stress and fluoxetine, an antidepressant, as specific molecular alterations that underlie the stress-mediated changes in social dominance. The AMPA-R phosphorylation in mPFC may serve as a biomarker of social dominance related to stress and depressive-like behaviors.

## Methods

### Animals

All animal experiments were approved by the Institutional Animal Care and Use Committee (IACUC) of the Korea Advanced Institute of Science and Technology (KAIST) and the Institute for Basic Science (IBS), and have been performed accordingly. All methods were performed in accordance with the relevant guidelines and regulations. Thirty mice were used in a set of experiments with three groups (CON, CRS, and CRS + FLU). Initially, mice were equally distributed (10 per group). However, one or two mice from each group could not be used because of the lower body weight or immobility. The final number of mice used in each test are given in the figure legends. Six-week-old male C57BL/6J mice were purchased from a local vendor (Orient Bio, Korea). Mice were housed in groups of five per cage for the behavioral experiments. Mice were stressed starting at 7 weeks of age. The mice were maintained at room temperature (22 °C ± 0.5 °C) with food and water available ad libitum under controlled conditions at a 12-h light/12-h dark cycle. For all animal behavior tests, the observers were blinded to the group assignments.

### Chronic restraint stress and drug treatments

Animals were randomly separated into control group (CON) or CRS group. The CRS group was randomly divided and received either normal saline solution (CRS) or fluoxetine (10 mg/kg; Sigma-Aldrich, St. Louis, Missouri) co-treatment (CRS + FLU). Fluoxetine was dissolved and diluted in normal saline solution, and administered by intraperitoneal injection 30 min before the restraint stress. CON mice received normal saline solution. For restraint stress, the mice were individually placed into 50-mL polypropylene conical tubes with a nose-hole for ventilation, and they were exposed to restraint stress (3 h/day) for 21 consecutive days. After restraint stress, mice were returned to the home cage. One day after the last day of stress, the mice were weighed and used for a series of behavioral assays.

### Animal behavioral tests

The mice were placed in the behavior room for 30 min for room habituation with white noise (65 dB). All behavioral tests were performed during the light cycle (09:00 to 17:00) in a dedicated sound-proof chamber with white noise (65 dB) and under a dim light (10 lux). The OFT, SI, and dominance tube test were performed with an EthoVision XT9 video tracking system (EthoVision Version 9, Noldus, Netherlands). TST and FST were conducted by two observers to minimize error.

### Open field test

The OFT was conducted based on our previous study^65^. Briefly, a mouse was placed in a white acrylic chamber (40 × 40 × 40 cm^3^) for 10 min to measure locomotor activity. An arena was designated as center zone (10 × 10 cm^2^). The mice were placed in a corner of the test box at the beginning of the test trial. During the test, the movement of mice in the chamber was recorded and automatically analyzed with EthoVision XT9 software. After every test, the chamber was cleaned with 75% ethanol.

### Tail suspension test

The TST was adapted from a method described by Steru *et al*.^66^. We used a customized tail suspension boxes (55 cm height × 30 cm width × 11.5 cm depth; material, Matte brown acryl). In order to prevent mice from interfering each other, each mouse was separated by a compartment (55 cm height × 15 cm width × 11.5 cm depth). Mice that climbed their tail or fell off the hanger were excluded from analysis. The immobility time was measured for 7 min.

### Forced swimming test

To measure immobility through the FST, we used the details of the paradigm from a previous report^67^. In brief, each mouse was placed in a Pyrex beaker (30 cm height, 16 cm diameter) containing 23 °C water with a depth of 17 cm. All mice were forced to swim for 6 min. The mice were habituated for the first 1 min, and the time of immobility was measured during the final 5 min.

### Sucrose preference test

To assess anhedonia, the SPT was adapted from a previous study^67^. The mouse cages were modified to fit two water bottles. One bottle was filled with water containing 1% sucrose, and the other was filled with pure water. For two consecutive days, the mice were acclimatized to the two-bottle conditions. After acclimation, the mice were tested for two additional days. On each test day, the fluid levels were noted. The position of the bottles was interchanged during testing days. The data are presented as the percentage of sucrose/total liquid consumption.

### Social interaction

For the SI test, the three-chamber test was performed as previously described^65^. The amount of time spent in each chamber was video recorded and automatically analyzed using the EthoVision XT system.

### Dominance tube test

Social dominance was measured using the tube test, which was adapted from Wang *et al*.^26^ The tube test apparatus is a Plexiglass tube with a length of 30 cm and an inside diameter of 2.6 cm. Small acrylic boxes (10 cm × 10 cm × 10 cm) were added to each end of the tube to facilitate the entry into the tube. For adaptation to the tube, each mouse was trained to run through the tube for three successive days. During the training trial, any highly anxious mice that were hesitant to enter the tube were not used for test trials. During the test trial, two mice with similar body weights from different groups were placed into opposite ends of the tube and guided to meet in the middle of the tube. A mouse that pushed the other mouse out of the tube was designated as the “winner” of that trial. During test trials, each trial progressed for 2 min, and each test mouse was challenged three times with a novel strange mouse from different groups (a match). For example, if a certain mouse won twice in succession, the test was stopped and the winner mouse got a score of 2, while the loser got a score of 0. If a mouse won in a first match, lost in a second match, and won again in the last match, the winner mouse got a score of 2, while the loser mouse got score of 1. Therefore, for the comparison between two groups in Fig. 3a, their winning points varied between 0 and 2. Total matches for each mouse were limited to four because of the body weight difference among groups. Therefore, for the correlation studies between behaviors and winning point of all three groups, winning point of each mouse varied between 0 and 4, providing an indication of the number of wins from a total of four matches per animal. Before each trial, the tube was cleaned with 75% ethanol.

### Determination of corticosterone level

After the last behavioral test, the mice were sacrificed to collect blood via cardiac puncture, and the serum was isolated and stored at −80 °C. The corticosterone level in serum was determined by a corticosterone enzyme-linked immunosorbent assay kit according to the manufacturer’s instructions (Cayman Chemical, Ann Arbor, MI).

### NBQX injection

NBQX (0.03 nmol/side) was administered unilaterally into either left or right prelimbic region (PL) in mouse brain 35 min before the dominance test. A Cannula tip (guide + dummy) was implanted to inject NBQX into the PL of mPFC. Saline infusion into PL was applied to the control group.

### Total protein extraction and western blot analysis

Based on the information from a mouse brain atlas (Paxinos and Franklin, 2007), the mPFC corresponding to ~1.9–2.7 mm anterior to the bregma, which contains the prelimbic and infralimbic cortices was dissected out from the mice using a Rodent Brain Slice Matrix (1 mm coronal intervals, ZIVIC Instruments). The tissue was homogenized with phosphate-buffered saline solution (PBS, Welgene, Korea) containing 0.32-M sucrose, 0.5-mM ethylenediaminetetraacetic acid, 0.5-mM ethylene glycol tetraacetic acid, 1% Triton X-100, 0.2% sodium dodecyl sulfate, protease inhibitor cocktail (Complete-EDTA free; Roche), and phosphatase inhibitor cocktail (PhosStop, Roche). The lysates were incubated in a refrigerator for 2 h and were centrifuged at 14,000 *g* for 15 min in 4 °C. The concentration of protein was determined using a bicinchoninic acid assay (Thermo Scientific, Waltham, MA). Thirty micrograms of protein were used for the western blot. The blots were then blocked in PBS including 1% bovine serum albumin and 0.1% Tween-20 for 1 h at room temperature and incubated with the antibodies at 4 °C overnight. All primary antibodies used in our study, except the GluA1-pS818 antibody, were purchased and used in the dilution of GluA1 (1:2000, NeuroMab, CA), GluA1-pS831 (1:2000, Millipore), GluA1-pS845 (1:2000, Cell Signaling, Danvers, MA), GluA2 (1:3000, Millipore), GluA2-pS880 (1:2000, Novus Biologicals, Littleton, CO), and β-actin (1:2000, Cell Signaling). The GluA1-pS818 antibody (1:1000) was made in a similar way to previous work^36^. The following day, the blots were washed and incubated with specific secondary antibodies using a horseradish peroxidase–linked goat anti-mouse IgG (1:10000, GenDEPOT, Barker, TX) and goat anti-rabbit IgG (1:10000, GenDEPOT) at room temperature for 1 h. The blots were developed with enhanced chemiluminescence (SuperSignal, Thermo Sciences) and imaged with an image capturing system (ChemiDoc XRS, Bio-Rad). For quantification of the western blots, the protein signals were measured and analyzed using ImageJ software (NIH, Washington, DC). To show the fidelity of our western blot analysis of AMPA-R phosphorylation, whole blots were presented as Supplementary Figures (Supplementary Figs S8 and S9).

### Statistical analysis

The statistical significance of differences among groups was assessed with Student’s *t*-test or one-way ANOVA. Linear regression was used to obtain a best-fit line for each plot. All data were presented as the mean ± standard error of the mean (SEM). A Bonferroni’s post hoc analysis was performed when p values were less than 0.05. All statistical analyses were performed using GraphPad Prism version 6.0 (La Jolla, CA).

## Electronic supplementary material

Supplementary Dataset 1–9

## Data Availability

All data generated or analyzed during this study are included in this published article (and its Supplementary Information files).
