# Supplementary material for: Stress-induced changes in social dominance are scaled by AMPA-type glutamate receptor phosphorylation in the medial prefrontal cortex
Source: Sci Rep. 2018 Oct 9;8:15008. doi: 10.1038/s41598-018-33410-1 (PMC6177388; doi:10.1038/s41598-018-33410-1)
Supplement: Supplementary file 1 — Supplementary Dataset 1–9 [file 41598_2018_33410_MOESM1_ESM.docx]

**Stress‐induced changes in social dominance scaled by AMPA‐type glutamate receptor phosphorylation in the medial prefrontal cortex** Min‐Jung Park, Bo Am Seo, Boyoung Lee, Hee‐Sup Shin, and Myoung‐Goo Kang

# igure 1

**Open field test**

- - 1. CON CRS CRS+FLU


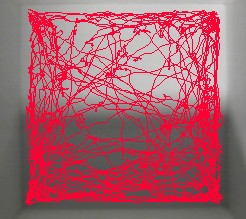

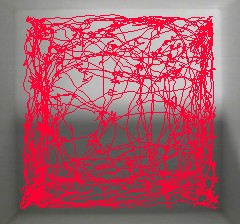

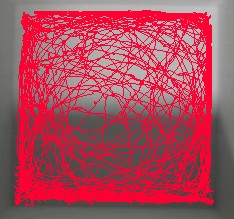


### c


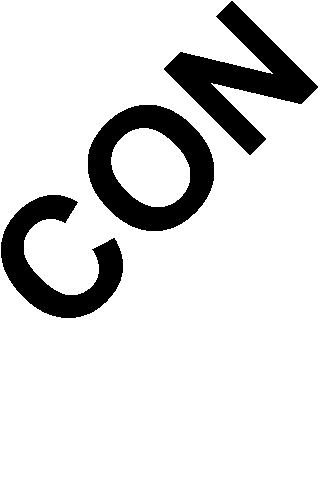

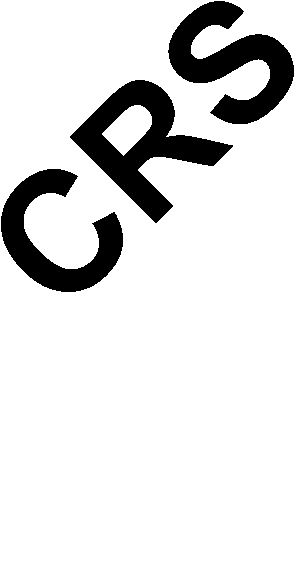

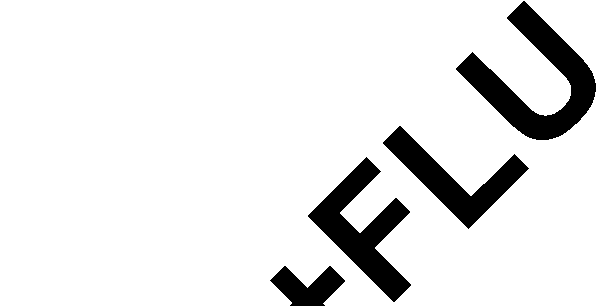

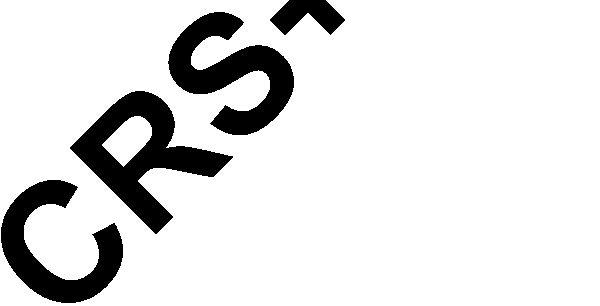

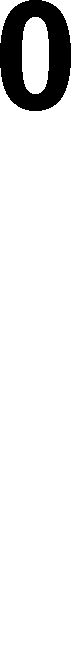

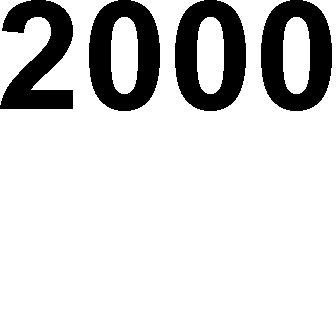

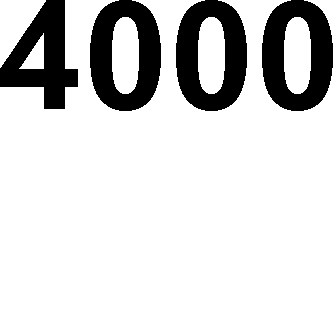

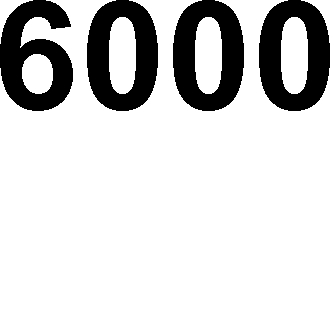

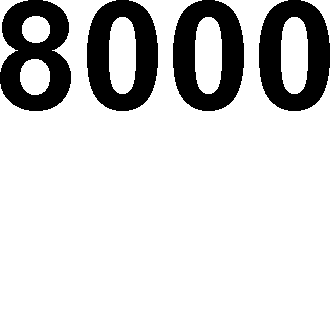

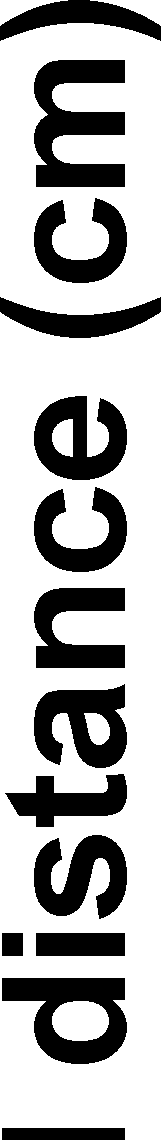

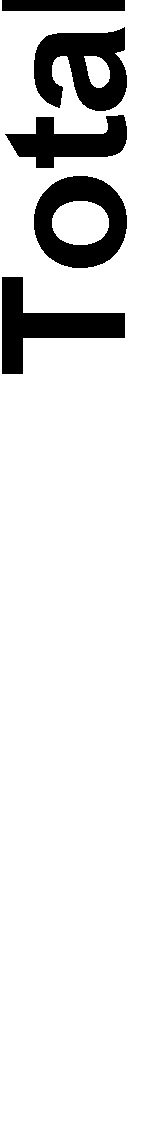

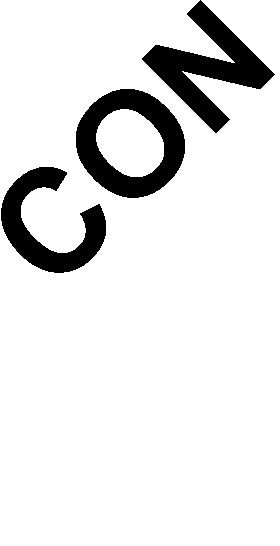

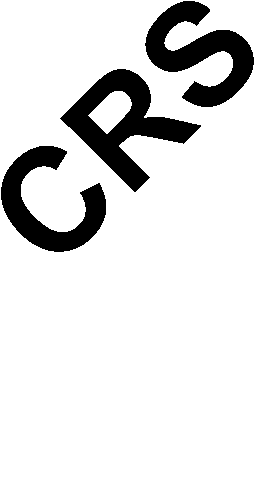

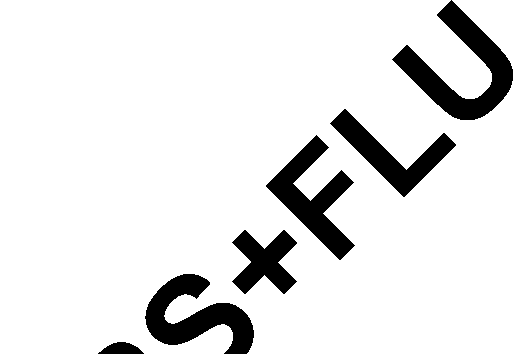

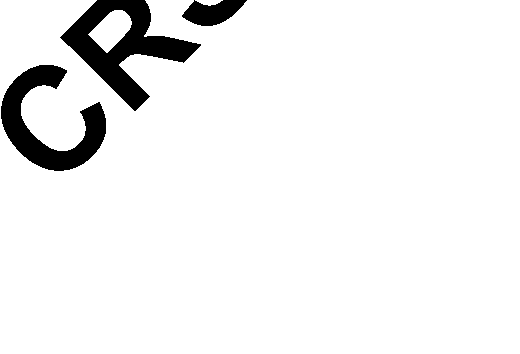

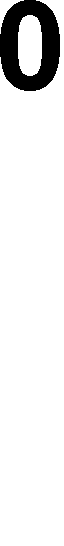

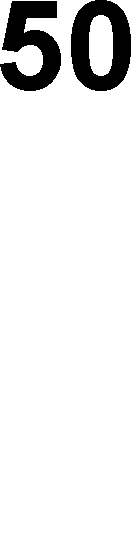

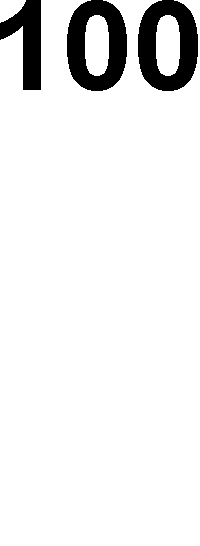

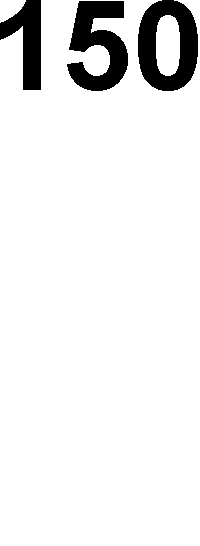

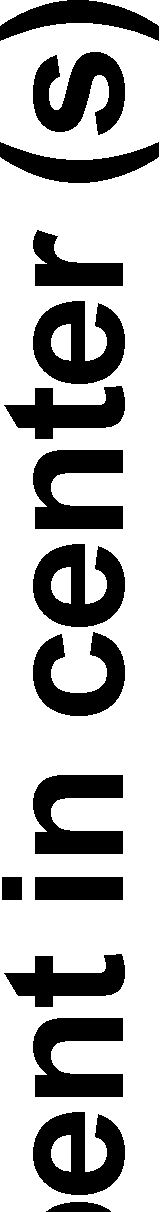

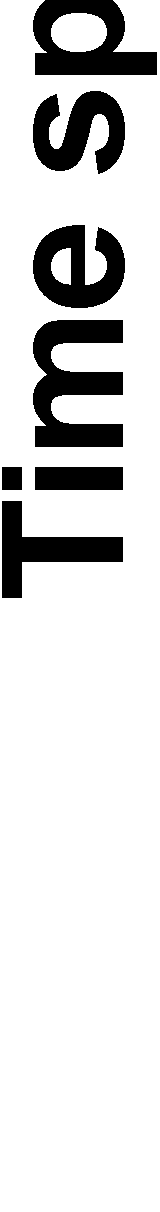


d

**Body weight**

**32**

* n.s


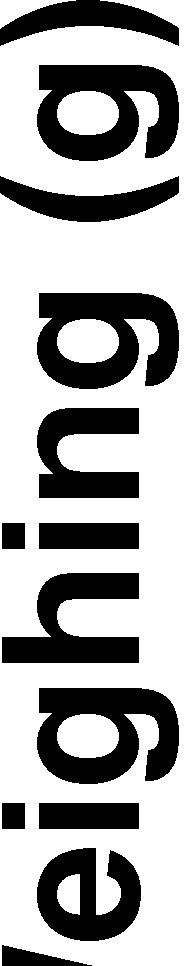

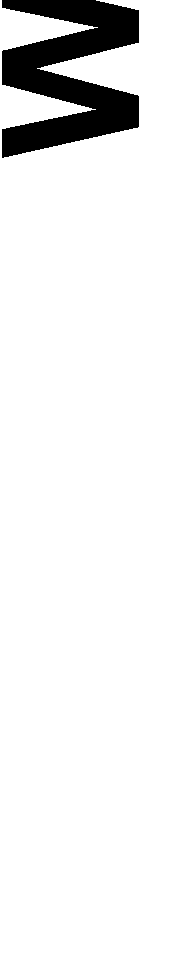


**30**

**28**

**26**

**24**

**22**

**20**

**Supplementary Figure 1**. Chronic restraint stress (CRS) had no effects on locomotion and anxiety behavior. (a) After CRS, mouse movement in open field was tracked and analyzed using EthoVision XT9. Trace in red color, center zone in black color. (b) No difference in total distant moved among three groups. One-way ANOVA with Bonferroni’s post hoc test, F(2,

27) = 0.1111, p = 0.8953. (c) No difference in time spent in center zone among three groups. One-way ANOVA with Bonferroni’s post hoc test, F (2, 27) = 1880, p = 0.1720 (d) Body weight was decreased by CRS. One-way ANOVA; F (2, 27) = 5.081, *p= 00134.

Post hoc Bonferroni’s multiple comparisons unveiled a significant difference between three groups. CON

(27.00±0.5416, n=10) vs CRS (24.80±0.4412, n=10),

*p = 0.0190; CRS vs CRS+FLU (24.93±0.4783, n=10),

p = 0.2499. Data shown are mean ± standard mean

error (SEM). OFT were measured during 10 min. Body weight was measured after termination of CRS.

### a b


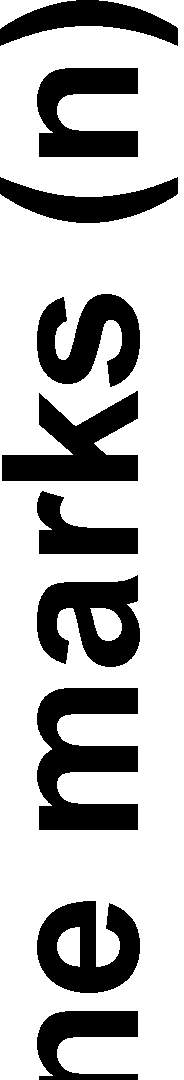

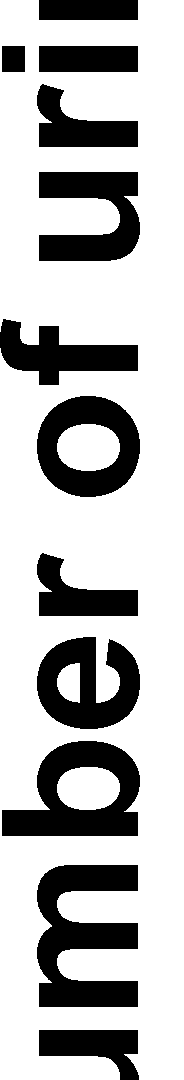

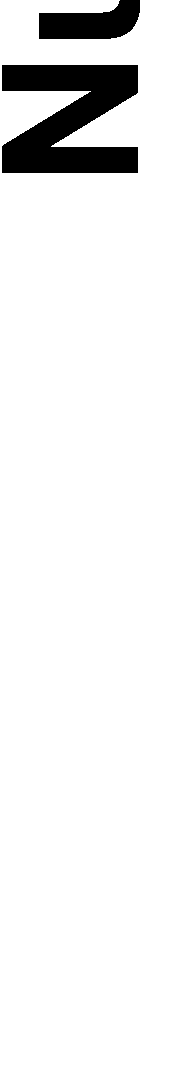

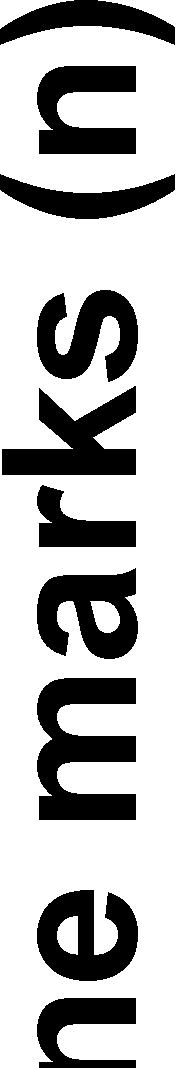

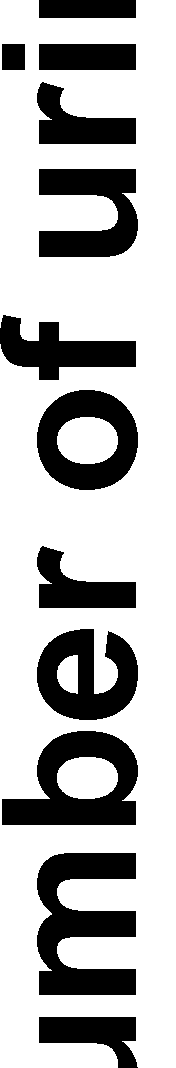

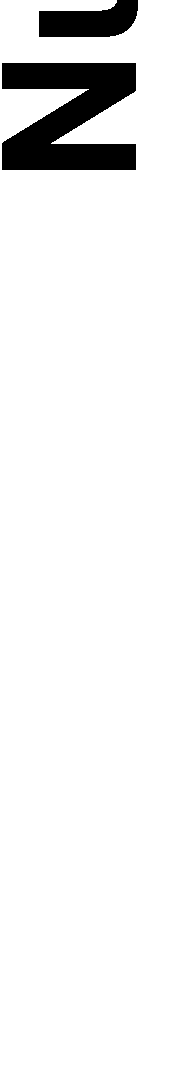


**80**


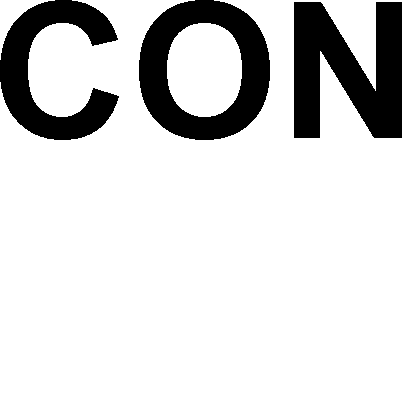

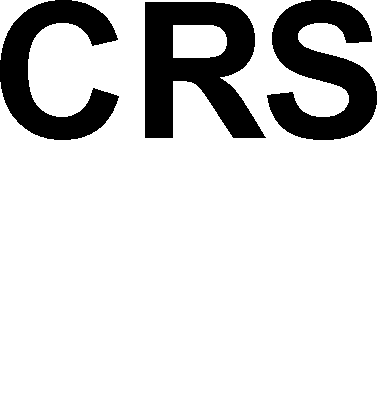

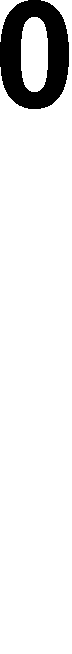

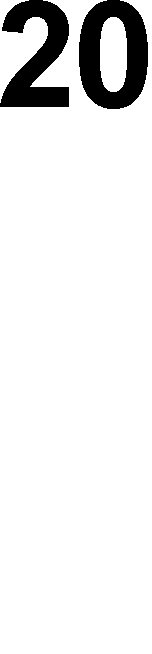

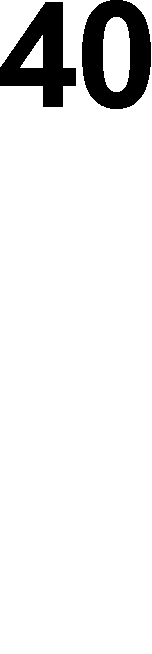

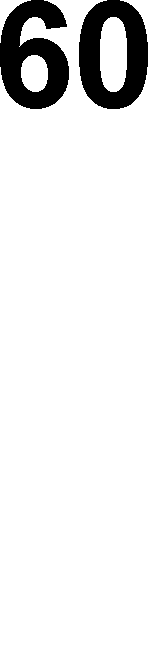

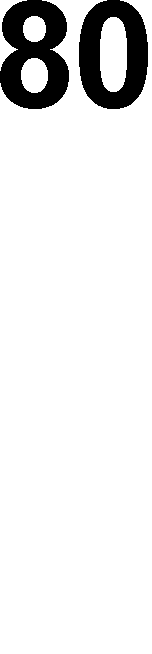


**

**60**

**40**

**20**

**0**

**le**

es

**CRS CRS+FLU**

**Supp mentary Figure 2**. The total number of

c squar withurinemarksduring20minexposure.


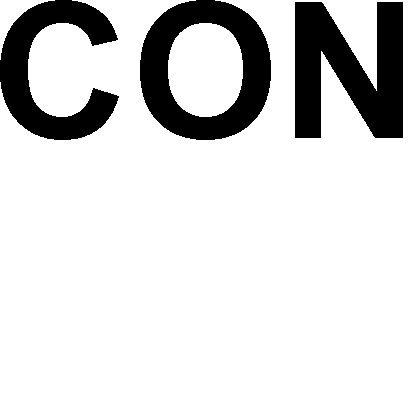

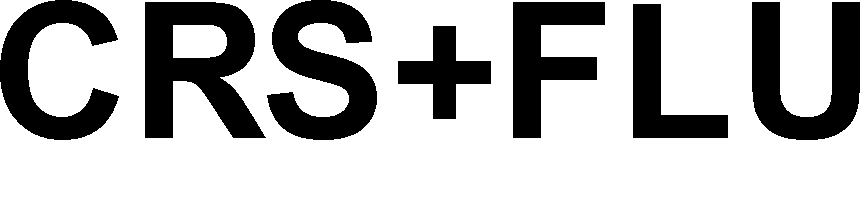

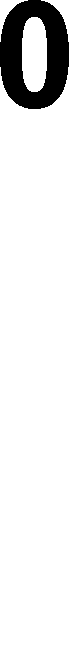

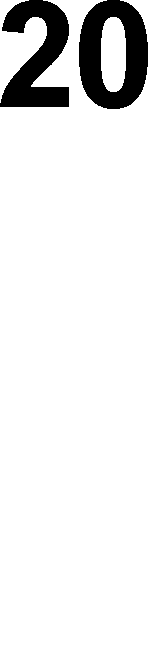

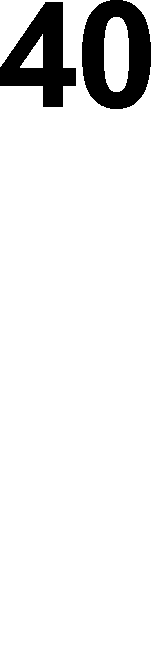

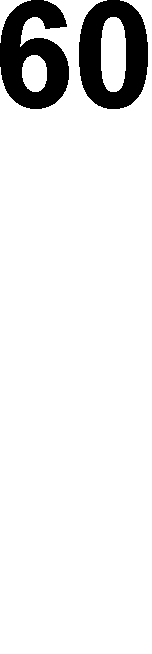

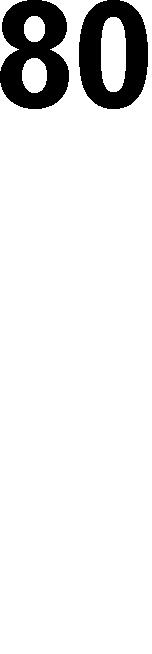


**Number of urine marks (n)**

1. The urine marks were decreased in CRS mice

compared to that in CON mice. CON (37.40±8.268, n=10) vs CRS (18.80±3.966,

n=10), *p=0.0489, Student’s paired t-test. (b) The urine marks were increased in the CRS+FLU group compared with that in the CRS group. CRS

(16.90±4.165, n=10) vs CRS+FLU (36.10±5.012,

n=10), **p=0.0036, Student’s paired t-test. (c) No significant difference of the urine marks between

CON and CRS+FLU groups. CON (43.90±5.085,

n=10) vs CRS+FLU (52.80±5.200, n=10),

Student’s paired t-test. Data are shown as mean ± SEM.

**Method:** The urine scent marking test was conducted as previously reported1-3. To test urine marking, mice were housed singly for 1 week before the test. For the three different groups of mice, 30 possible pairs of urine marking were tested. The filter paper was placed in an empty polycarbonate cage (28 × 13 × 17 cm) to collect urine, and each cage was divided into two equal-sized compartments by a transparent partition with holes permitting animals to see and smell each other but preventing physical contact. Each pair of mice was placed on opposite sides of a partition for 20 min, and allowed to scent mark on the paper substrates. After the end of each session, the mice were moved to their home cages. The marked sheets of filter paper were treated with ninhydrin spray (LC-NIN-16; TritechForensics Inc., Southport, NC, USA) and dried for 24 h, which allowed visualization of the urine marks as purple spots. To analyze the urine marks, the number of scent marks was measured by placing a transparent grid (each 1 × 1 cm) over the dried substrate paper. The total number of grids that contained scent marks was counted.

Reference:

1. Wang, F. et al. Bidirectional control of social hierarchy by synaptic efficacy in medial prefrontal cortex. Science 334, 693-697, doi:10.1126/science.1209951 (2011).
2. Kim, S. et al. Deficiency of a brain-specific chemokine-like molecule, SAM3, induces cardinal phenotypes of autism spectrum disorders in mice. Sci Rep 7, 16503, doi:10.1038/s41598-017-16769-5 (2017).
3. Arakawa, H., Arakawa, K., Blanchard, D. C. & Blanchard, R. J. Scent marking behavior in male C57BL/6J mice: sexual and developmental determination. Behav Brain Res 182, 73-79, doi:10.1016/j.bbr.2007.05.007 (2007).

### a

**1.4**


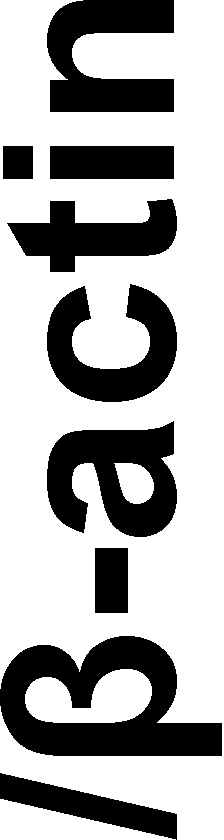

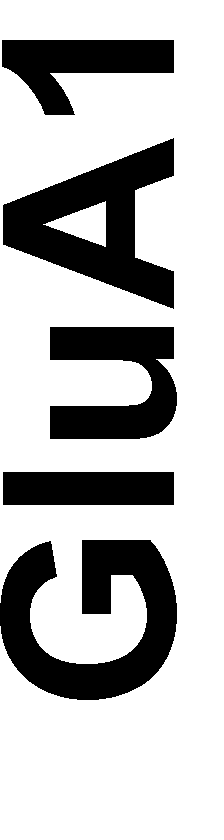

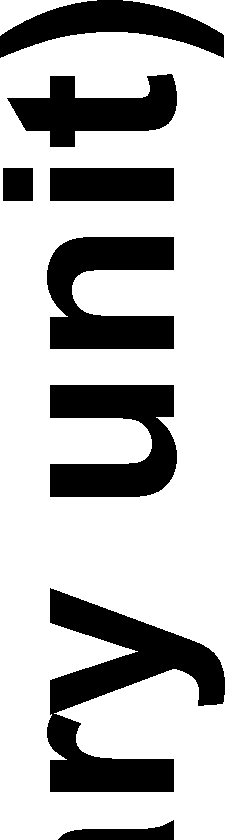

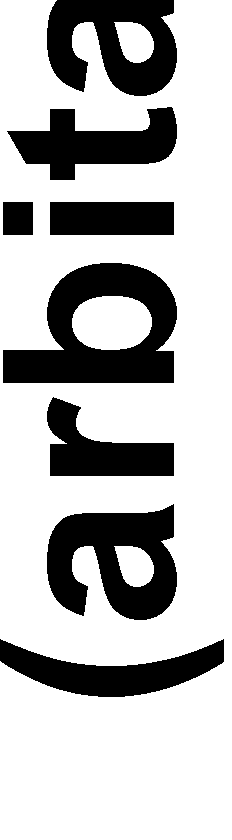


**1.2**

**1.0**

**0.8**

**0.6**

### c

**GluA2/β-actin**

**(arbitary unit)**

b

##### GluA1

**pS845/GluA1**

**(arbitary unit)**

**R2 = 0.70**

**p = 0.074**

**0 1 2 3 4**

###### winning point

d


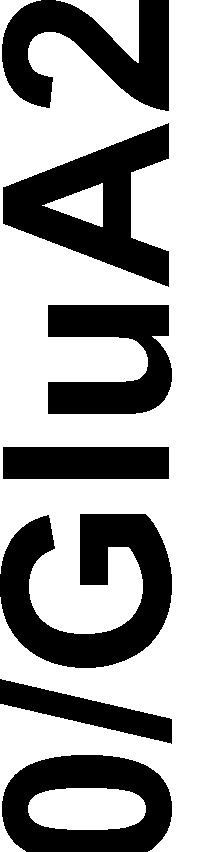

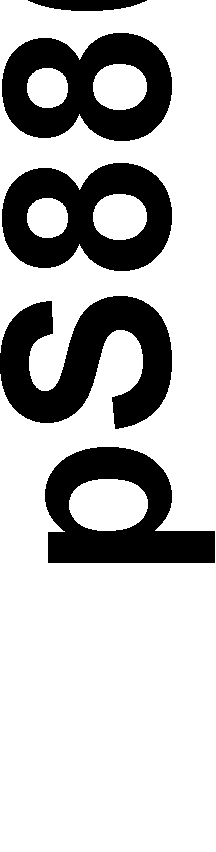

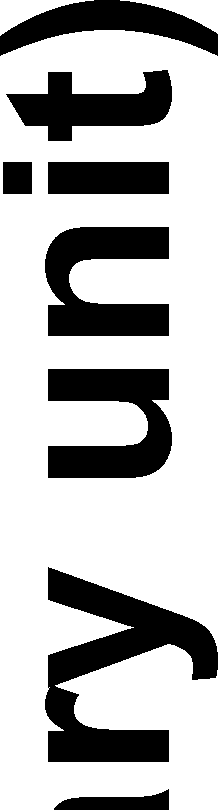

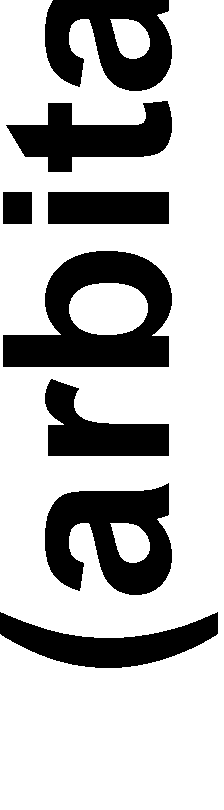

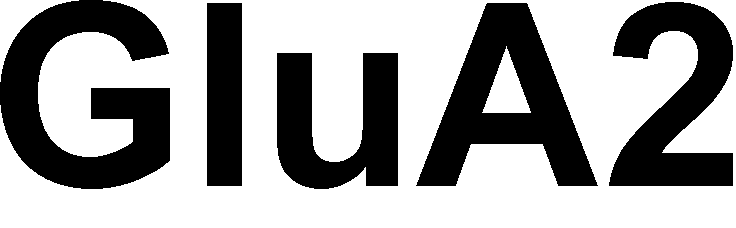

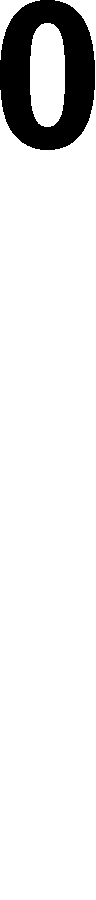

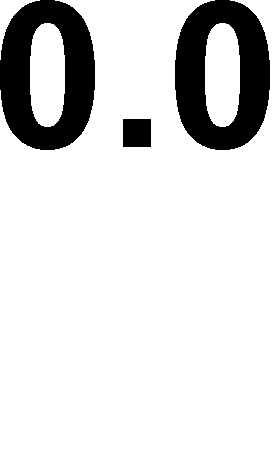

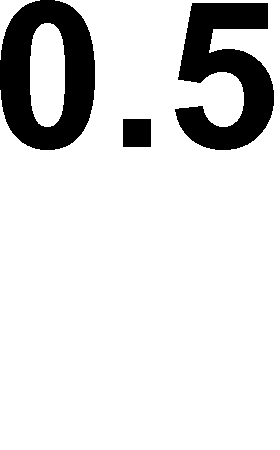

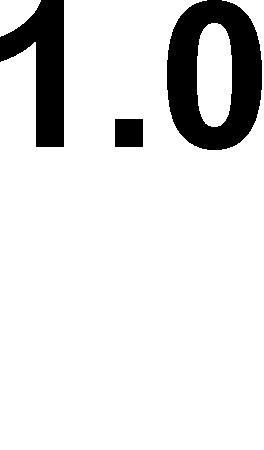

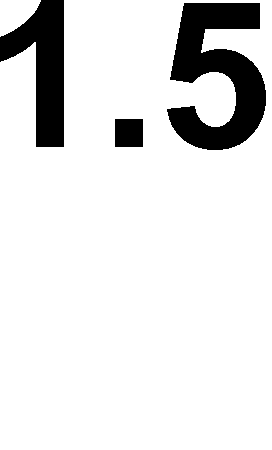


**R2 = 0.20**

**p = 0.448**


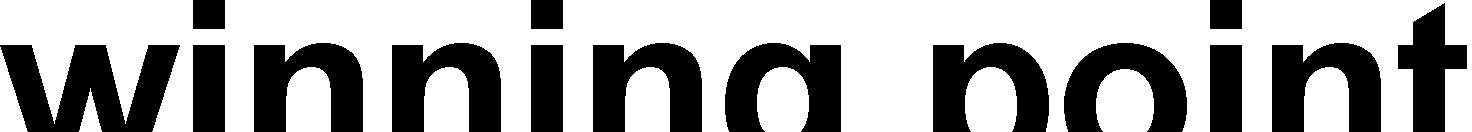

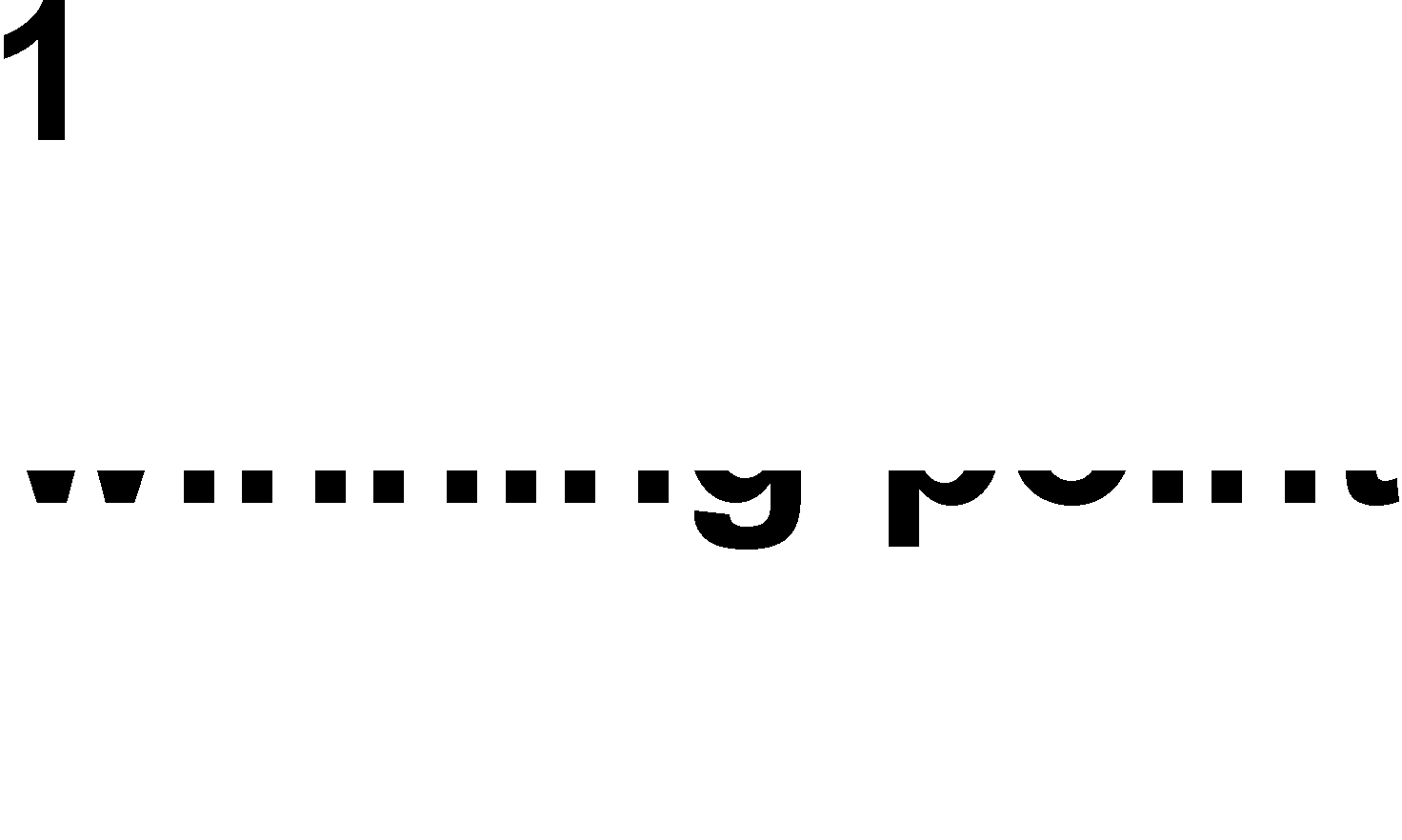

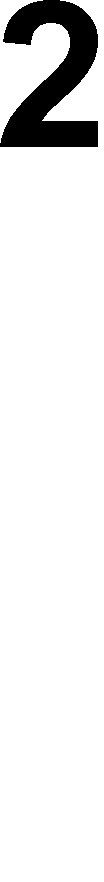

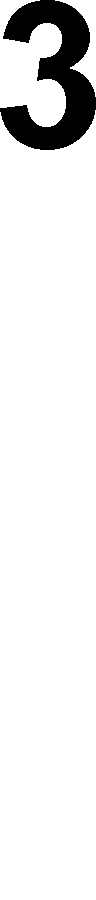


**1.5**

**1.0**

**0.5**

**0.0**

#### pS845

**R2 = 0.39**

**p = 0.258**

**0 1 2 3 4**

###### winning point


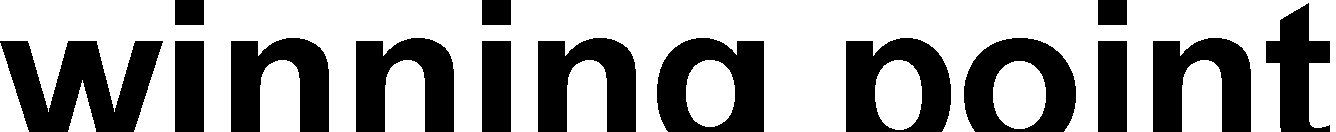

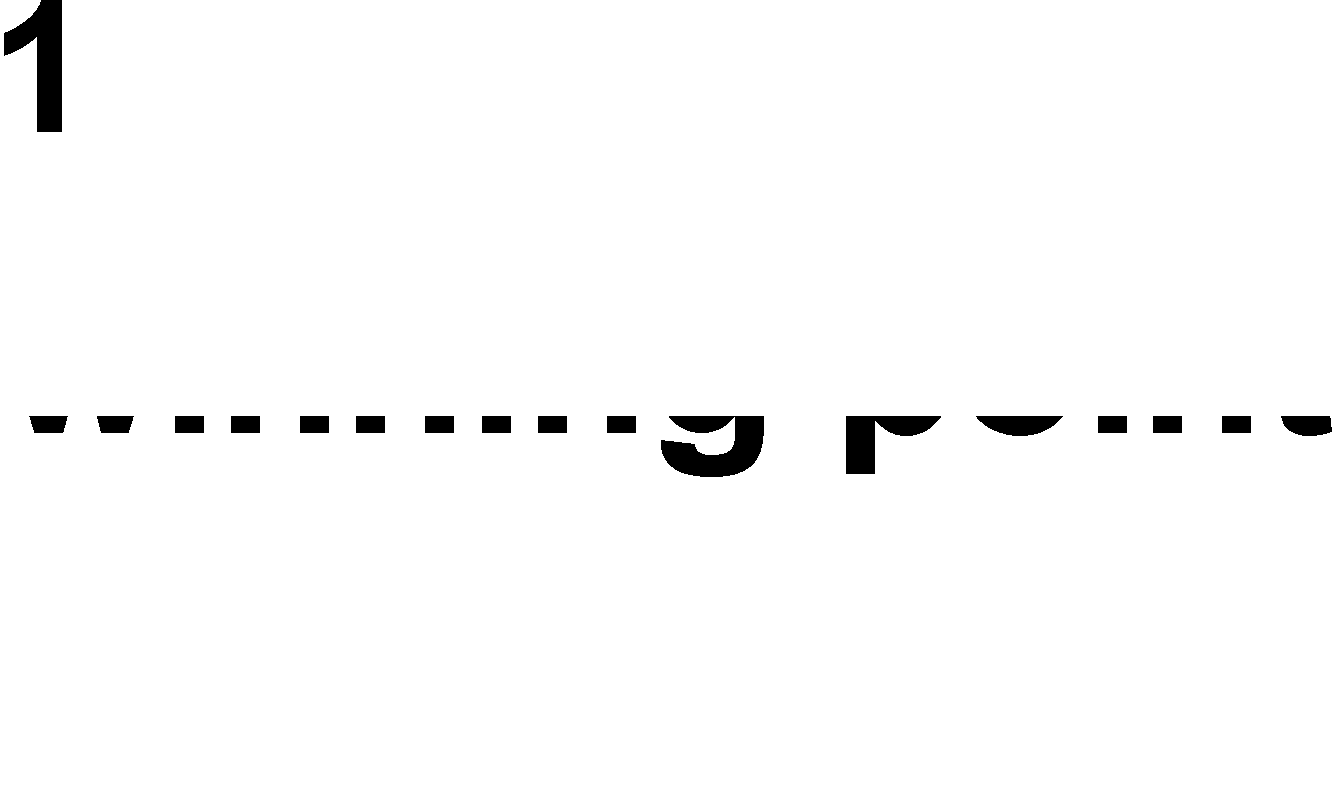

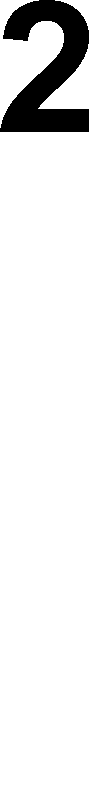

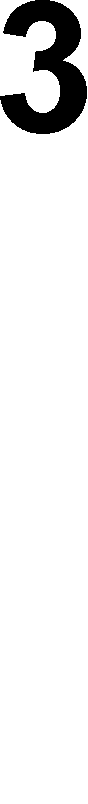

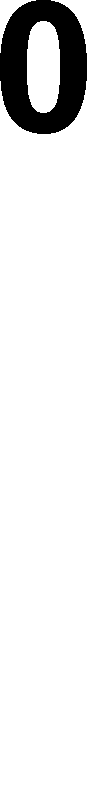

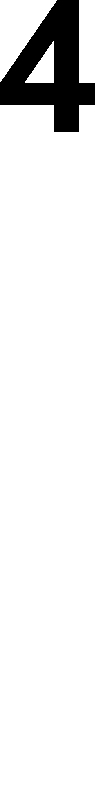

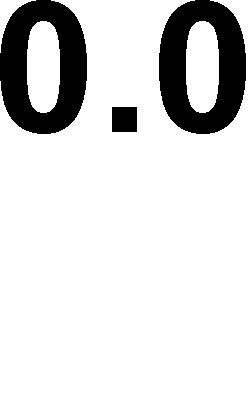

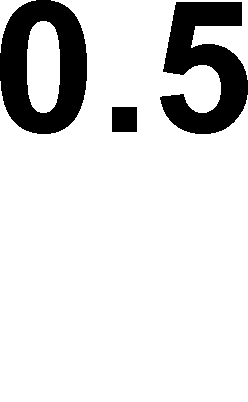

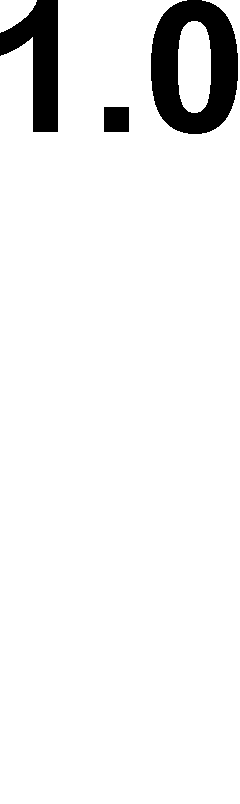

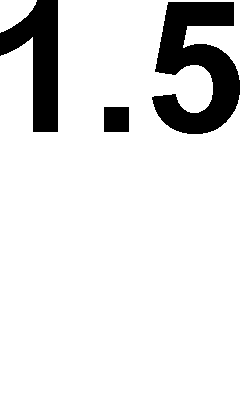

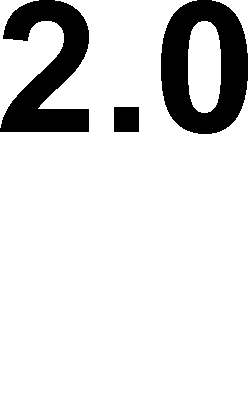

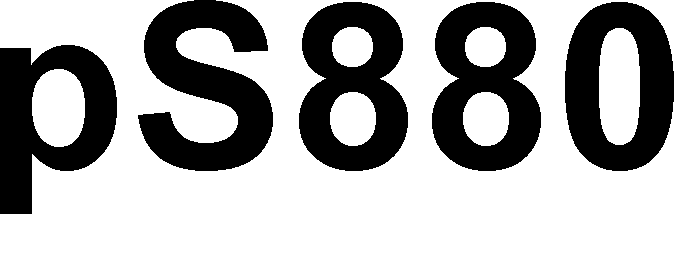


**R2 = 0.72**

**p = 0.069**


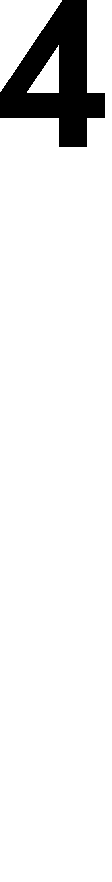


**Supplementary Figure 3**. The correlation of AMPA-R subunits or their phosphorylation with social dominancy. No significant correlation of winning point in tube test with GluA1 (R2 = 0.70, n = 15, p = 0.074) (a), pS845 (R2 = 0.39, n=15, p = 0.258) (b), GluA2 (R2 = 0.20, n =

15, p = 0.448) (c) and pS880 (R2 = 0.72, n = 15, p = 0.069) (d).Data shown as mean ± SEM.

### a b


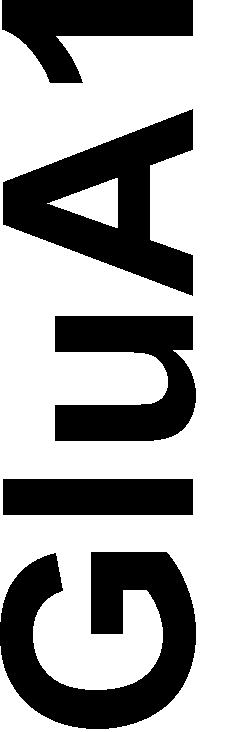

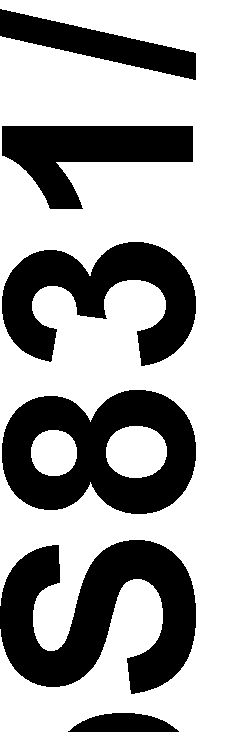

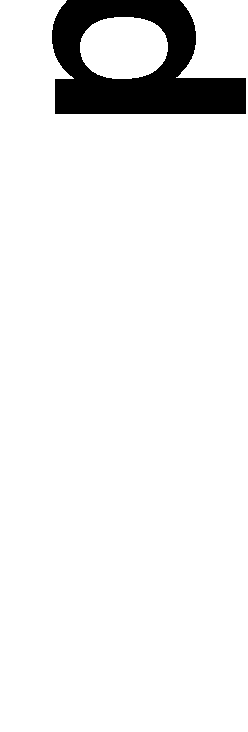

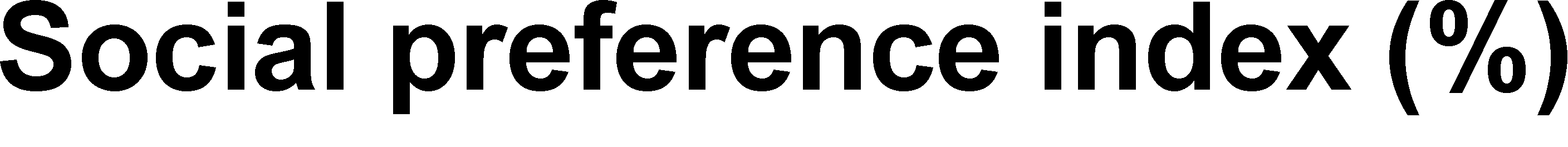

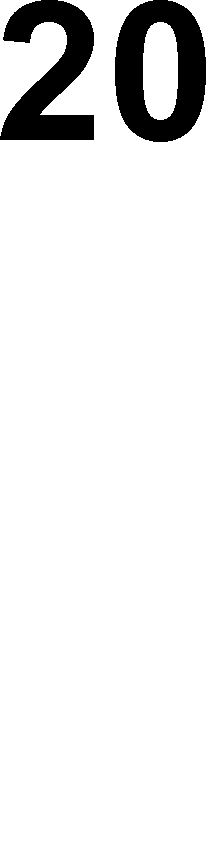

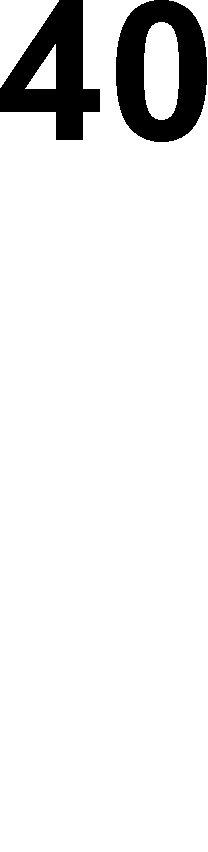

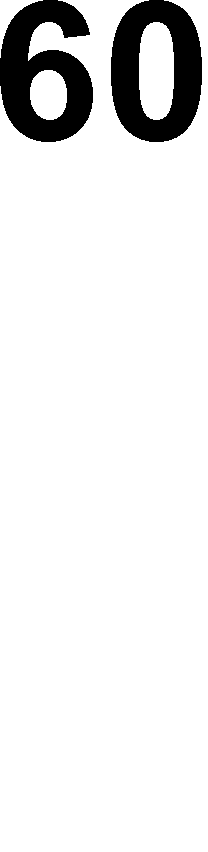

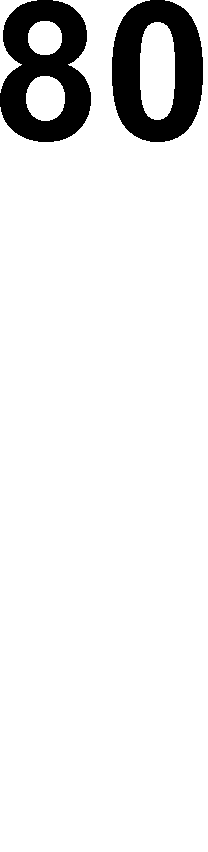

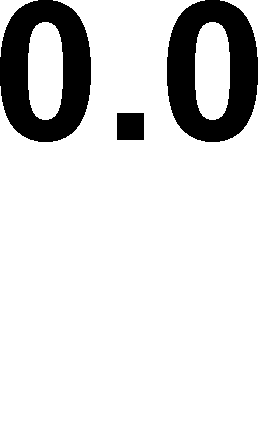

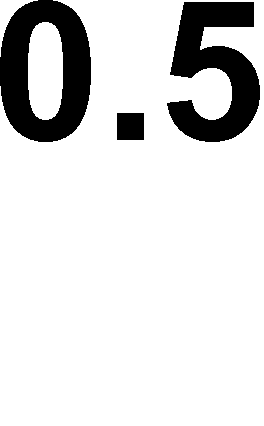

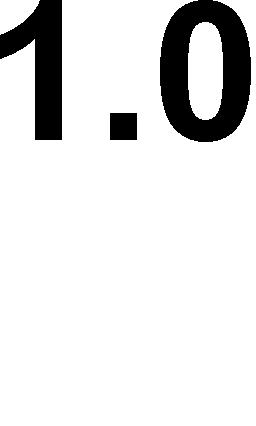

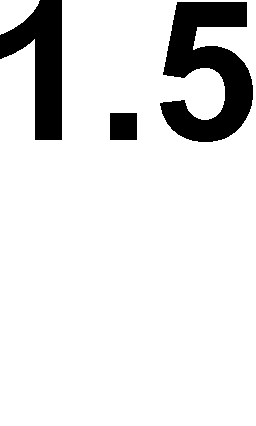

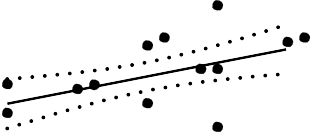

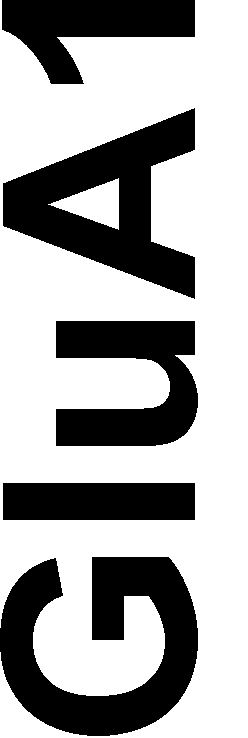

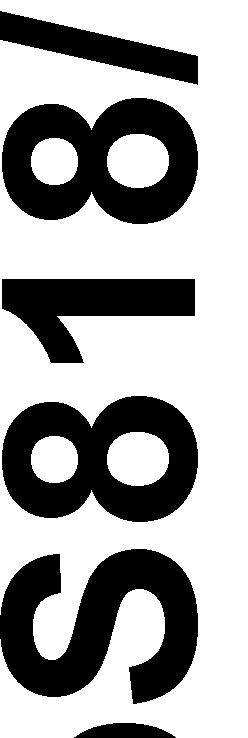

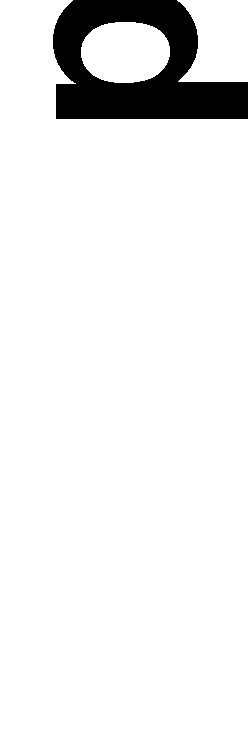

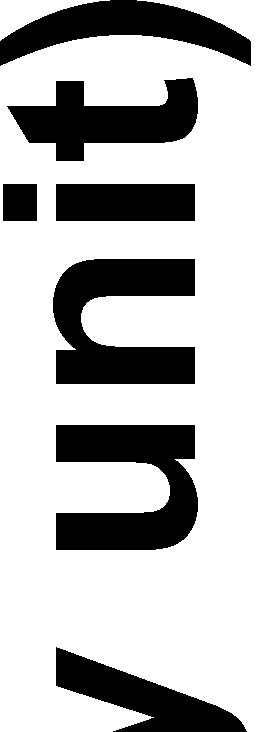

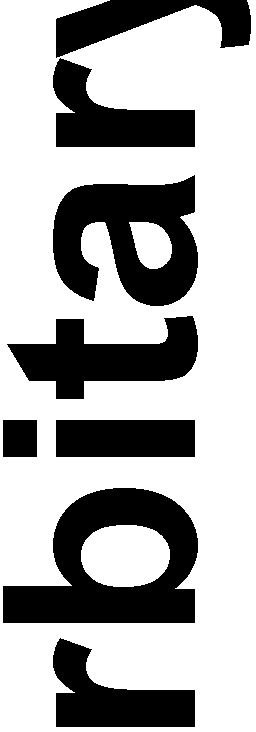

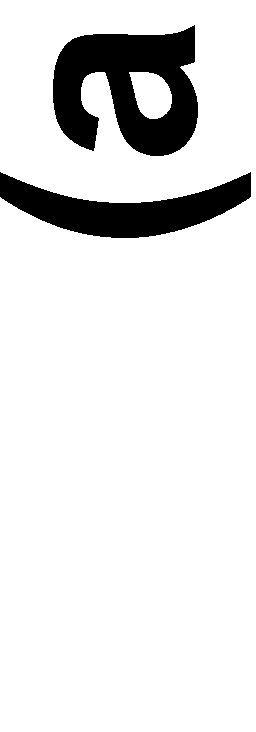

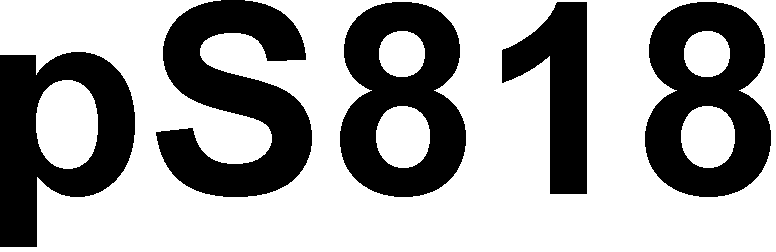


**R2 = 0.8533**

***p = 0.0250**

### c d


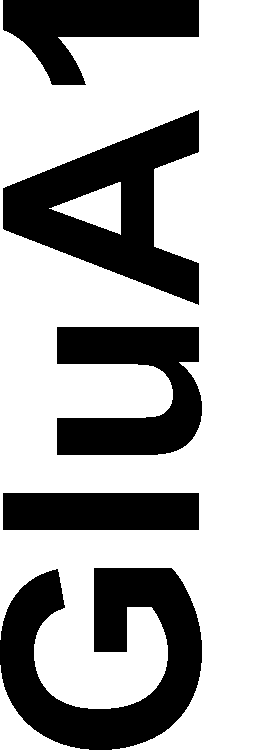

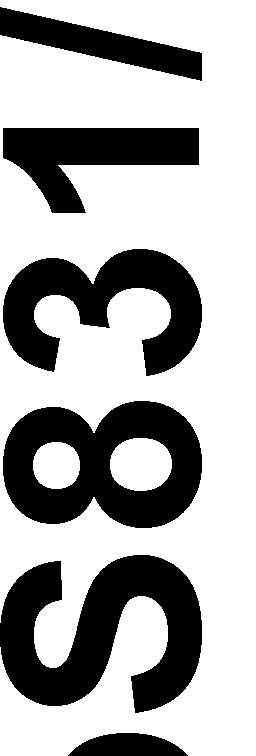

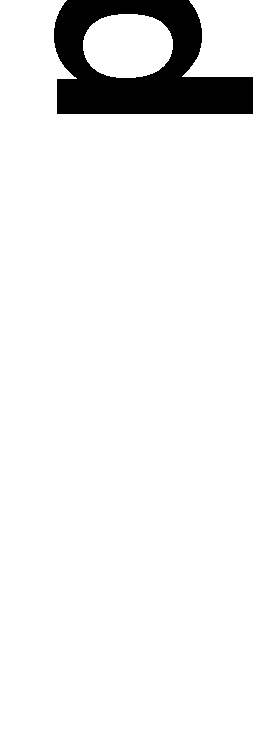


**R2 = 0.6878**

**p = 0.1707**

**R2 = 6616**

**p = 0.0942**

**R2 = 0.9899**

****p = 0.0050**

**Supplementary Figure 4**. The correlation between phosphorylation of pS818 or pS831 and social interaction indexes. (a) Significant correlation of social preference index with pS818 (R2 = 0.8533, n = 14, *p = 0.0250). (b) No significant correlation of social preference index with pS831. (c) No significant correlation of social novelty index with pS818. (d) Significant correlation of social novelty index with pS831(R2 = 0.9899, n = 14,

**p = 0.0050). Data shown as mean ± SEM.

Tail suspension test

Dominance tube test

*

*

Force swimming test

*

a

b

c

**Supplementary Figure 5**. Fluoxetine altered depressive-like behaviors and dominance. (a) In the Tail suspension test (TST), immobility was significantly lower in the FLU group (fluoxetine i.p. for 21 days) compared with the CON group (saline i.p. for 21 days) group (n = 15, 15, Student’s *t*-test, **p*<0.05). (b) In the Force swimming test (FST), immobility was significantly lower in the FLU group compared with the CON group (n = 15, 15, Student’s *t*-test, **p*<0.05). (c) In tube test, the winning points of the FLU group were significantly higher compared with those of the CON group (n = 15, 15, Student’s *t*-test, **p*<0.05). Winning points were calculated as described at Figure 3 legend and main text.

**pS831**

**GluA1**

**β-actin**

**pS818**

**pS845**

**GluA2**

**pS880**

**CON**

**FLU**

*

*

a

b

c

d

e

f

g

**Supplementary Figure 6**. Phosphorylation of AMPA-R was altered by fluoxetine. (a) Representative data from western blot analyses of AMPA-R subunits and their phosphorylation in the mouse mPFC. (b-g) Quantification of the western blot analyses of AMPA-R subunits and their phosphorylation demonstrated: significant increase of the serine phosphorylation (pS) on 845 of GluA1 and significant increase of the pS on 880 of GluA2 by fluoxetine. (b) Quantification of the western blot analyses of pS818 (n=10, 10, Student’s *t*-test, P= 0.831). (c) Quantification of the western blot analyses of pS831 (n=10, 10, Student’s *t*-test, P= 0.485). (d) Quantification of the western blot analyses of pS845 (n=10, 10, Student’s *t*-test, **p*< 0.05). (e) Quantification of the western blot analyses of GluA1 (n=10, 10, Student’s *t*-test, P= 0.257). (f) Quantification of the western blot analyses of pS880 (n=10, 10, Student’s *t*-test, **p*< 0.05). (g) Quantification of the western blot analyses of GluA2 (n=10, 10, Student’s *t*-test, P= 0.287).

a

b

c

+1.98 from bregma

*

**Supplementary Figure 7**. The dominance was decreased by unilateral inactivation of AMPA-R in mPFC regardless of fluoxetine. (a) In tube test, the winning points of NBQX group (injected with 0.03 nmol/side) were lower than those of the control group (CON) (n=9,9; Mann–Whitney rank-sum test, T=108.5, U=17.5, **P*<0.05). (b) Fluoxetine administration (FLU, 10 mg/kg i.p.) did not affect the NBQX effect on dominance. In tube test, the winning points of FLU + NBQX group were lower than those of the CON group (n=9,9; Mann–Whitney rank-sum test, T=106.5, U=19.5, *P<0.05). (c) Representative histology of an mPFC cannula placement.

**Method**: Fluoxetine (10 mg/kg i.p) was administered once a day for 21 days. NBQX (0.03 nmol/side) was administered into the prelimbic region (PL) 35 min before the test. Cannula tip (guide + dummy) was implanted to inject NBQX into the PL of mPFC. Black circles indicate the targeting point of individual mouse. Infusions were unilaterally performed but were bilaterally represented to show being administrated into either left or right side. Saline infusion into PL was applied for CON.

*

**Supplementary Figure 8**. Raw data from Figure 4. Whole western blots are presented

a

b

c

d

**CON**

**CRS**

**CRS+FLU**

**pS818**

**pS818**

**(kDa)**

200

150

100

75

**pS831**

**CON**

**CRS**

**CRS+FLU**

**pS831**

200

150

100

75

**(kDa)**

**pS845**

**CON**

**CRS**

**CRS+FLU**

**pS845**

200

150

100

75

**(kDa)**

**CON**

**CRS**

**CRS+FLU**

**GluA1**

**GluA1**

200

150

100

75

**(kDa)**

**Supplementary Figure 9**. Raw data from Figure 4. Whole western blots are presented

a

b

c

**CON**

**CRS**

**CRS+FLU**

**pS880**

**pS880**

200

150

100

75

**(kDa)**

**GluA2**

**CON**

**CRS**

**CRS+FLU**

**GluA2**

200

150

100

75

**(kDa)**

**β-actin**

**CON**

**CRS**

**CRS+FLU**

**β-actin**

75

50

**(kDa)**
